# Gαi1/3 Are Essential for BMP2‐Induced Osteogenesis by Bridging BMP Receptors to the Gab1 Signaling Complex

**DOI:** 10.1002/advs.76837

**Published:** 2026-08-10

**Authors:** Huajian Shan, Chaowen Bai, Yifei Zhu, Chenyang Xu, Sonngtao Li, Qize Sun, Chang She, Jinyu Bai, Cong Cao, Yitian Yang, Xiaozhong Zhou

**Affiliations:** ^1^ Department of Orthopedics The Second Affiliated Hospital of Soochow University Suzhou Jiangsu China; ^2^ Clinical Research Center of Neurological Disease Institution of Neuroscience Second Affiliated Hospital of Soochow University Soochow University Suzhou China; ^3^ Department of General Surgery Comprehensive Breast Health Center Ruijin Hospital Shanghai Jiao Tong University School of Medicine Shanghai China; ^4^ Department of Anesthesiology and Perioperative Medicine Henan Provincial People's Hospital People's Hospital of Zhengzhou University Zhengzhou China

**Keywords:** BMP2, fracture, Gab1, Gαi1/3, osteogenesis, signaling

## Abstract

BMP2 serves as a pivotal regulator of skeletal development, homeostasis, and repair. Previously, we demonstrated that Gαi1/3 (G protein subunit alpha i1/3) is not only involved in G protein‐coupled receptor (GPCR) signal transduction but also acts as a key mediator of tyrosine kinase receptor signaling. However, its role in bone formation and BMP2 signaling remains elusive. In this study, we report that the expression of Gαi1/3 is significantly downregulated in the bone tissue of patients with senile osteoporosis. Gαi1/3 double knockout mice exhibited delayed skeletal development and reduced bone mass. Mechanistically, Gαi1/3 is required for BMP2 signal transduction, as its deletion impaired BMP2 downstream signaling. In BMSCs (bone marrow–derived mesenchymal stem cells), Gαi1/3 knockout suppressed BMP2‐induced osteogenesis, while overexpression enhanced it. In the femoral fracture model, knockdown of Gαi1/3 in BMSCs reduced the ability of BMP2 to promote fracture healing. Furthermore, in neonatal mice with conditional knockout of Gαi1/3 in BMSCs, skeletal development was delayed. Gab1 is indispensable for Gαi1/3‐mediated BMP2 signaling, as Gαi1/3 binds to both the BMP2 receptor and Gab1 to mediate BMP2 biological function. Collectively, our findings reveal the critical role of the Gαi1/3‐Gab1 axis in BMP2 signaling and osteogenesis, providing potential therapeutic targets for osteoporosis and bone repair.

## Graphical abstract

The Gαi1/3 protein is a crucial connecting protein closely related to the signal transduction process of bone formation‐related cytokines such as BMP2. Gαi1/3 mediates downstream signaling pathways such as Smads and MAPK by binding to adaptor proteins like Gab1, thereby promoting the osteogenic effect of BMSCs. Deficiency of either Gαi1/3 or Gab1 will inhibit the signal transduction of osteogenic factors and bone formation.

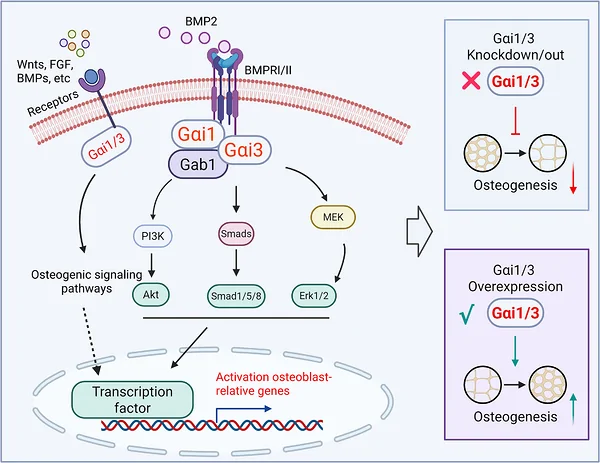

## 1 Introduction

Bone formation is a fundamental physiological process crucial for maintaining skeletal integrity and systemic homeostasis [1]. It ensures the normal development of the skeletal system, supports body structure, protects internal organs, and maintains calcium and phosphorus homeostasis [1]. Adequate osteogenic activity prevents senile osteoporosis, significantly improves the quality of life in the elderly, and facilitates bone regeneration and repair after fracture [2]. With advances in regenerative medicine and molecular biology, targeted therapies to enhance bone formation have emerged as a pivotal strategy for managing bone injury repair and degenerative bone diseases [3, 4]. This approach holds significant clinical and public health value by addressing unmet needs in conditions such as osteoporosis and fractures. Bone formation is primarily driven by BMSCs and encompasses sequential stages, including osteogenic differentiation, osteoid secretion, and mineralization [1, 2]. These processes are regulated by multiple signaling factors. BMPs induce the differentiation of BMSCs into osteoblasts [5]. The Wnt/β‐catenin pathway promotes osteogenic proliferation and inhibits adipogenesis [6]. TGF‐β regulates bone matrix synthesis [7]. Working synergistically, cytokines promote bone formation, which is vital for skeletal development, fracture repair, and the treatment of metabolic bone diseases. In particular, the BMP family plays a pivotal role in osteogenesis [5].

BMP2 (Bone morphogenetic protein 2), a key signaling molecule in the TGF‐β superfamily, regulates multiple biological processes, including osteogenesis and tissue repair [8]. Given its vital role in bone development, maintenance, and regeneration, BMP2 signaling has emerged as a major focus in bone regeneration research [9]. Mechanistically, BMP2 promotes osteogenesis by stimulating the proliferation and differentiation of bone marrow mesenchymal stem cells (BMSCs) and osteogenic precursor cells [10]. In clinical practice, rhBMPs serve as effective bone‐inducing agents for the treatment of skeletal defects [11]. At the molecular level, BMP2 signaling relies on the activation of the intracellular serine/threonine kinases of type I and type II BMP receptors [12]. Ligand‐binding events induce the transcription of multiple osteogenesis‐associated genes through the activation of a complex array of downstream intracellular mediators that include the PI3K‐Akt‐mTORC1, Erk‐MAPK, and the Smad pathways [13, 14]. However, the underlying signaling mechanisms of BMP2‐induced osteogenesis have not yet been fully elucidated.

Among inhibitory G protein alpha subunits (Gαi), three primary subtypes exist: Gαi1, Gαi2, and Gαi3 [15]. Upon activation by G protein‐coupled receptors (GPCRs), Gαi proteins inhibit adenylyl cyclase (AC), thereby reducing intracellular cAMP levels [15]. Our prior studies have demonstrated that Gαi1 and Gαi3 critically regulate non‐canonical RTKs (receptor tyrosine kinases) signaling by modulating both PI3K‐Akt‐mTOR and Erk pathways. This mechanism is conserved across multiple RTKs, including EGFR [16], BDNF receptor TrkB [17], and VEGF receptor VEGFR2 [18].

Following RTKs stimulation by the binding of growth factors, Gαi1 and Gαi3 are required for the activation of downstream PI3K‐Akt‐mTOR and Erk signal transduction [16, 17]. In human cells, BMP type I/II receptors constitute a rare class that possesses intrinsic serine/threonine kinase activity [14]. Whether Gαi mediates the signal transduction of receptor serine/threonine kinases remains unknown. In this study, we observed that Gαi1/3 is downregulated in the bone tissue of elderly osteoporosis patients, with its expression level correlating with bone formation capacity. Mechanistically, we demonstrate that Gαi1/3 is essential for BMP2 signal transduction, primarily by interacting with BMP2 receptors (BMPR1A/B and BMPR2) and Gab1. Consequently, loss of Gαi1/3 expression or function significantly impairs BMP2‐mediated bone formation and compromises fracture healing. These findings underscore that Gαi1/3 extends beyond its traditional biological functions of regulating RTK signaling. Crucially, it also serves as a key mediator of downstream signaling for BMP2, a receptor serine/threonine kinase. As a promising therapeutic target for enhancing bone formation and treating fractures, Gαi1/3 provides the foundation for novel treatment strategies.

## 2 Methods

### 2.1 Study Approval

Human studies were approved by the Ethics Committee of the Second Affiliated Hospital of Soochow University, and all participants provided written informed consent. All animal protocols were reviewed and approved by the Animal Ethics Committee of Soochow University (Approval ID: SUDA20181015A05) and conducted in compliance with institutional guidelines. Mice were housed under specific pathogen‐free (SPF) conditions at a controlled temperature of 22–24°C under a 12‐h light/dark cycle, with ad libitum access to water and standard chow. All experimental procedures were performed in accordance with international ethical guidelines and relevant national regulations [19].

### 2.2 Reagents and Antibodies

Recombinant human BMP2 was sourced from Calbiochem, and puromycin was acquired from Sigma‐Aldrich. Cell culture media and supplements were obtained from Gibco BRL. The following primary antibodies were purchased from Santa Cruz Biotechnology: tubulin, Erk1/2, Akt1/2, GSK3β, SHP2, Shc, Gab1, Grb2, Gαi1, Gαi2, and Gαi3. Goat anti‐rabbit IgG‐HRP and goat anti‐mouse IgG‐HRP secondary antibodies were also obtained from Santa Cruz Biotechnology. The β‐actin monoclonal antibody (mouse) was supplied by Sigma. Additional phospho‐specific and signaling antibodies, including p‐Akt (Ser473/Thr308), p‐Smad1/5, p‐Gab1 (Tyr627), p‐S6K (Thr389), p‐S6 (Ser235/236), p‐GSK3α/β (Ser21/9), p‐Erk1/2 (Thr202/Tyr204), p‐MEK1/2, Runx2, BMPR1A, and BMPR1B, were procured from Cell Signaling Technology.

### 2.3 Murine Bone Marrow Mesenchymal Stem Cell Isolation and Culture

BMSCs were harvested from 6–8 week‐old C57BL6/J mice following established protocols [20]. Tissue Preparation: Euthanized mice were sterilized with 70% ethanol, and femurs were aseptically dissected into DMEM (Biological Industries) supplemented with 100 U/mL penicillin/streptomycin (P/S). Marrow Extraction: After removing soft tissues and trimming the femoral ends, the bone marrow was flushed using a 27‐gauge needle with growth medium (α‐MEM supplemented with 10% FBS and P/S). The cell suspension was filtered through a 70‐µm cell strainer (BD Falcon) and plated in 100‐mm dishes. Adherence Selection: Non‐adherent cells were removed after 3 h. The medium was replaced every 12 h for 72 h, and then every 48 h until reaching 70% confluence. Passaging: Cells were washed with PBS, detached using 0.25% trypsin/0.02% EDTA for 5 min at room temperature (RT), and expanded for subsequent experiments.

### 2.4 Cell Culture

BMSCs from young (average age 27.7 years) or older (average age 71.3 years) patients with fracture were extracted and induced osteogenic differentiation. BMSCs were maintained in DMEM (1×) supplemented with 10% FBS, 2 mm L‐glutamine, 100 mg/L sodium pyruvate, and 1% penicillin/streptomycin, and cultured at 37°C with 5% CO_2_. MC3T3‐E1 osteoblastic cells were grown in high‐glucose DMEM with 10% FBS and 1% penicillin/streptomycin under identical incubation conditions. For both cell types, the medium was replaced every 24 h during the initial 48 h to remove non‐adherent cells, followed by changes every 2–3 days. Osteogenic differentiation was induced by culturing cells in growth medium supplemented with 100 mg/L sodium pyruvate, 50 µm ascorbic acid, and 10 mm β‐glycerophosphate for 14 days [20].

Mouse embryonic fibroblasts (MEFs)‐including wild‐type (WT), Gαi1/Gαi3 double knockout (DKO), Gαi1/Gαi2/Gαi3 single knockout (SKO), and Gab1 KO MEFs—were propagated in DMEM with 10% FBS. Prior to experiments, MEFs were serum‐starved (0.5% FBS, overnight) and equilibrated in warm PBS for 30 min [21].

### 2.5 Alkaline Phosphatase (ALP) and Alizarin Red S Staining

BMSCs cultured in 6‐well plates were processed as follows. Fixation: Cells were rinsed three times with PBS and fixed with 4% paraformaldehyde for 15–30 min. Alkaline Phosphatase (ALP) Activity: After washing twice with PBS, the fixed cells were incubated with a p‐nitrophenyl phosphate substrate (Alkaline Phosphatase Assay Kit, Beyotime, #C3206) for 2 h at room temperature in the dark, according to the manufacturer's protocol. Mineralization Detection: Calcium deposits were stained using Alizarin Red S (Solarbio, #G3280) following the manufacturer's instructions.

### 2.6 Co‐Immunoprecipitation

Cells were transfected with the indicated plasmids using the SuperKine Lipo3.0 Efficient Transfection Reagent (Abbkine Scientific Co., Ltd.). Following transfection, cells were lysed, and the resulting lysates were subjected to immunoprecipitation with specific antibodies conjugated to magnetic beads using the Pierce Crosslink Magnetic IP/Co‐IP Kit (Thermo Fisher Scientific). The immunoprecipitates were collected, washed three times with lysis buffer, and subsequently analyzed by Western blotting.

### 2.7 qPCR, Western Blotting and Others

Total RNA was isolated from BMSCs using TRIzol reagent (Thermo Fisher Scientific) according to the manufacturer's protocol. cDNA was synthesized using the PrimeScript RT Master Mix (Takara). RT‐qPCR amplification was performed with TB Green Premix Ex Taq (Tli RNaseH Plus) on a Roche LightCycler 480 II system. Western blotting, co‐immunoprecipitation (Co‐IP), and confocal microscopy were conducted as previously described [22]. For Western blotting, 40 µg of total protein was loaded per lane. To detect different target proteins, identical lysate samples were run in parallel on separate gels (this procedure was applied to all figures).

### 2.8 Micro‐Computed Tomography (Micro‐CT) Analysis

Micro‐CT scanning and analysis were performed using the NEMO Micro‐CT system (Model NMC‐100). Mouse femurs were positioned between the X‐ray source and the CMOS detector and rotated 360° along the vertical axis for projection images. Images captured by the CMOS detector were processed using image analysis software with a mineralization threshold set at 350 mg/cm^3^ calcium hydroxyapatite equivalent. The scanning parameters were set to a voltage of 90 kV, a current of 0.04 mA, and a scan duration of 20 min. The volume of interest for trabecular bone analysis, located 1000 µm from the growth plate and extended a further 1500 µm longitudinally in the proximal direction region of interest (ROI). Image reconstruction was conducted with Avatar software utilizing the Feldkamp–Davis–Kress (FDK) algorithm, achieving a pixel size of 0.012 mm. Quantitative evaluations included bone mineral density (BMD), bone volume per tissue volume (BV/TV), trabecular thickness (Tb.Th), trabecular number (Tb.N), and trabecular spacing (Tb.Sp). For cortical bone, the region of analysis was 5% of femoral length in the femoral mid‐diaphysis for measuring cortical thickness (Ct.Th).

Femurs were dissected free of soft tissues and scanned using a SkyScan 1172 micro‐CT system (Bruker, Belgium) at 9 µm isotropic voxel size (50 kV, 500 µA, 0.5° rotation step). Analyze the regions of interest (ROI) of femoral fractures in mice, such as the central area of callus (500 µm at each end of the fracture line). Quantitative analysis of the fracture callus tissue images was performed using CTAn software (version 1.9, Sky Scan). The following parameters were tested: callus bone mineral density (BMD, g/cm^3^), callus volume/total volume (CV/TV, %), callus number (1/mm), and callus volume (mm^3^). CV, TV, CV/TV, and BMD of each specimen were recorded for analysis [23].

### 2.9 Animal Surgery

A classic mouse femoral fracture model was applied in this study [23]. Six‐week‐old male mice were divided into different groups. First, the mice were anesthetized with isoflurane. The skin was incised from the outside of the right knee to the greater trochanter side. A 23G needle was inserted into the bone marrow cavity of the mouse's femur and broke the middle part of the femur. X‐rays were taken following fracture to exclude any mice with unsatisfactory fractures. According to the method used in a previous study [24], 100 µL of free BMP2 (500 ng/per mouse) or PBS was injected into the fracture site. Analgesia was achieved by a single subcutaneous injection of a buprenorphine sustained‐release preparation (1 mg/kg). At the conclusion of the experiment, mice were euthanized via rapid anesthesia and death induced by hypoxia in a sealed container filled with CO_2_.

The sample size was determined based on preliminary data from the evaluation of bone volume fraction (BV/TV), the primary outcome measure. Before conducting the formal experiment, we performed a preliminary exploratory study and observed a relatively consistent trend in treatment effects with low intra‐group variability. In the preliminary experiments, we observed a significant effect size (Cohen's d = 1.8) and a within‐group standard deviation (SD = 0.1278). Based on these findings, we performed an a priori sample size estimation using G*Power 3.1 software. Test type: Two‐sample independent t‐test (two‐tailed); α error probability: 0.05; statistical power (1‐β): 0.80; effect size d: 1.8. The calculation results showed that a minimum sample size of 5 animals per group is required to achieve 80% statistical power. In addition, in high‐quality publications in the field of osteoporosis research, the typical sample size ranges from 5 to 7 mice per group [25, 26, 27]. In accordance with the “3R” principles of animal research (Reduction, Replacement, Refinement), the number of animals used should be minimized while still satisfying statistical requirements.

### 2.10 Bioinformatics Studies

Publicly available scRNA‐seq data (GSE150703) from 1.5‐ and 16‐month‐old mice were analyzed using Seurat (v3.0). Key steps included: Data Processing:1) Quality‐controlled raw counts. 2) Identified top 5,000 variable genes (FindVariableFeatures). 3) Reduced dimensions using PCA (top 30 PCs). Cluster Analysis: (1) Unsupervised clustering (FindClusters). (2) UMAP visualization. (3) Cluster annotation using markers from GeneCards. Downstream Analysis: (1) Density plots for Gαi1/Gαi3 expression. (2) Osteoblast‐specific DEG analysis (up‐/down‐regulated genes). (3)Top 10 enriched pathways (BP & KEGG).

### 2.11 Molecular Docking Prediction

Structure Acquisition: (1) Experimental structures: Obtained from the Protein Data Bank. (2) Predicted structures: Generated using AlphaFold. Structure Preparation: (1) Processed with AutoDockTools‐1.5.7. (2) Removal of water molecules. (3) Addition of polar hydrogens. Docking Analysis: (1) Performed using the GRAMM web server (protein‐protein docking). (2) Complex optimization. (3) Manual refinement of docking results. (4) Repeat water removal and hydrogen addition. Interaction Analysis: (1) Protein‐protein interaction prediction. (2) Visualization of complexes using PyMOL [28].

### 2.12 Alcian Blue/Alizarin Red Double Staining

Postnatal day 3 mouse specimens were fixed in 4% paraformaldehyde (PFA, Solarbio, P1110) at 4°C for 24 h, dehydrated in graded ethanol, cleared in xylene, and embedded in paraffin. Sagittal sections (5 µm) were cut using a Leica RM2016 microtome. For staining, sections were deparaffinized, rehydrated, and incubated with 0.1% Alcian Blue 8GX (#C0155, Beyotime) in 3% glacial acetic acid for 24 h at room temperature. After washing with acidic ethanol (1% HCl in 70% ethanol), sections were counterstained with 0.5% Alizarin Red (Sigma‐Aldrich, #A5533) in 0.1% ammonium hydroxide for 10 min to visualize calcium deposits, followed by brief differentiation in 0.1% KOH (15 s). GAG specificity was verified by hyaluronidase pretreatment (10 mg/mL, 37°C, 2 h). Imaging was performed with an OlyVIA slide scanner using brightfield and spectral unmixing.

### 2.13 Histology and IHC

Following 48‐h fixation in 10% formalin and 2‐week decalcification in 10% EDTA, femurs and tibiae were paraffin‐embedded and sectioned at 5 µm using an HM 355S rotary microtome (ThermoFisher). For morphological assessment, longitudinal coronal sections (femurs) and transverse sections (tibiae) were prepared, while fracture analysis utilized femoral coronal sections. Deparaffinized sections underwent histochemical staining (H&E and Masson's) and immunohistochemical analysis according to established protocols [31]. Primary antibodies targeting Runx2 (CST #12556S), Osterix (Abcam #ab209484), and OPN (Proteintech #22952‐1‐AP) were applied overnight at 4°C, with subsequent detection using an HRP‐streptavidin system (Dako) and hematoxylin counterstaining. Stained sections were imaged by light microscopy (Leica) and quantitatively analyzed using ImageJ software.

### 2.14 Calcein‐Alizarin Red Double Labeling Procedure

Calcein and Alizarin Red stock solutions (10 and 20 mg/mL, respectively) were prepared in distilled water or PBS (pH 7.4), aliquoted, protected from light, and stored at 4°C. Working solutions (1 mg/mL calcein, 2 mg/mL Alizarin Red) were prepared in a diluent containing 0.9% NaCl and 2% NaHCO3, warmed to body temperature, and filtered for sterilization. For in vivo labeling, mice received intraperitoneal injections of calcein (5 mg/kg) 10 days before sacrifice, followed by Alizarin Red (5 mg/kg) 3 days before sacrifice. Femurs were harvested, fixed in 4% paraformaldehyde, and processed without decalcification. Tissues were dehydrated, embedded in resin, and sectioned longitudinally. Sections were protected from light and imaged using fluorescence microscopy. The distance between the centers of the green (calcein) and red (Alizarin Red) fluorescent bands was measured using image analysis software to calculate dynamic bone formation parameters.

### 2.15 Generation of Gαi1/3 DKO and Col1a1‐Cre; Gαi1/3fl/fl Mice

Gαi1/3 double knockout (DKO) mice were created using CRISPR‐Cas9 technology as we previously reported [18]. Mice with conditional knockout of Gαi1/3 in the osteoblast lineage (Col1a1‐positive cells; Gαi1/3^OB−^): The Col1a1‐Cre mice were purchased from Jiangsu Jicui Yaokang Biotechnology Co., LTD. Gαi1/3^fl/fl^ mice were obtained from Jackson Laboratory (Sacramento, CA, USA). We crossed Gαi1/3^fl/fl^ mice with Col1a1‐Cre transgenic mice to generate Col1a1‐Cre; Gαi1/3^fl/+^ progeny, which were used for subsequent mating to produce homozygous Col1a1‐Cre; Gαi1/3^fl/fl^ mice.

### 2.16 Femoral Metaphysis Injection of AAV

Adult C57BL/6 mice (6–8 weeks old, 23.5–25.2 g) were anesthetized, and intramedullary injection of the virus into the femur was performed as previously described. Adeno‐associated virus serotype 9 (AAV9) vectors harboring Gαi1 or Gαi3 shRNA sequences under the control of the osteoblast‐specific Runx2 promoter were constructed. AAV injections (0.1 µl per mouse) were administered following a previously reported protocol [21].

### 2.17 Gαi1/3 shRNA

Following seeding at 1 × 10^5^ cells/well in 6‐well plates, MEFs/MC3T3‐E1/BMSCs were transduced with lentiviral particles encoding Gαi1‐ or Gαi3‐specific shRNAs [17, 18]. After 48 h, the medium was replaced with puromycin‐containing medium (refreshed every 48 h) and maintained for 10–12 days. Knockdown efficiency exceeding 90% was validated at both the protein (Western blot) and mRNA (qPCR) levels.

### 2.18 CRISPR/Cas9 Knockout of Gαi1 and Gαi3

CRISPR/Cas9 lentiviral constructs for Gαi1 and Gαi3 knockout were acquired from Genechem (Shanghai, China) [18], and the constructs were selected with puromycin after being transfected into cells. Control groups received non‐targeting sgRNA (Santa Cruz Biotechnology). Successful Gαi1/3 knockout was validated through Western blot and quantitative PCR analysis.

### 2.19 Gαi1/3 Overexpression

Cells were seeded in 6‐well plates at a density of 1 × 10^5^ cells/well before adenoviral transduction (Ad‐Gαi1, Ad‐Gαi3, or empty vector control). Following puromycin selection, transgene expression was validated by Western blotting and qPCR at the protein and mRNA levels, respectively [17].

### 2.20 Statistical Analysis

Data are presented as mean ± SD. The sample size for each experiment was determined based on preliminary data or prior studies in the field to ensure adequate statistical power (typically aiming for >80% power and α = 0.05). Statistical analyses were performed using GraphPad Prism 8 software. Prior to parametric testing, the normality of data distribution was assessed using the Shapiro‐Wilk test, and the homogeneity of variances was assessed using the Brown‐Forsythe test or F test, as appropriate. For comparisons between two groups, unpaired two‐tailed Student's t‐test was used when data met the assumptions of normality and equal variance; otherwise, the nonparametric Mann‐Whitney U test was applied. For comparisons among three or more groups, one‐way ANOVA followed by Tukey's post hoc test was used when data met the assumptions of normality and homogeneity of variances. If the assumptions for ANOVA were violated, the Kruskal‐Wallis test followed by Dunn's post hoc test was employed. All experiments were repeated at least three times independently. ns, *p* > 0.05. ^∗^ *p* < 0.05; ^∗∗^ *p* < 0.001; ^∗∗∗^ *p* < 0.001. A *p*‐value of < 0.05 was considered statistically significant. Randomization and blinding were adopted to reduce bias.

## 3 Results

### 3.1 Gαi1 and Gαi3 Participate in Osteogenic Function and Signaling, and Expression Is Reduced in Aging Osteoblasts or BMSCs

Loss of Gαi1/3 affects the formation of bone and cartilage in mice [29]. To investigate the potential role of Gαi1/3 in osteogenesis, we analyzed single‐cell RNA sequencing data (GSE150703) from the bone marrow of 1.5‐ and 16‐month‐old mice, identifying 16 distinct cell clusters (Figure 1A). We found that the number of osteoblasts and chondrocytes in the bone marrow of 16‐ month‐old mice was significantly reduced compared with 1.5‐ month‐old mice, while adipocytes were significantly increased (Figure 1B). Differential expression analysis revealed that Gαi1/3 levels were significantly reduced in the osteoblasts of 16‐month‐old mice (Figure 1C), an age associated with diminished osteogenic capacity in males [30]. To validate this age‐related decline, we examined Gαi1/3 expression in bone marrow mesenchymal stem cells (BMSCs), confirming that Gαi1/3 levels are lower in aged mice (Figure 1D).

**FIGURE 1:**
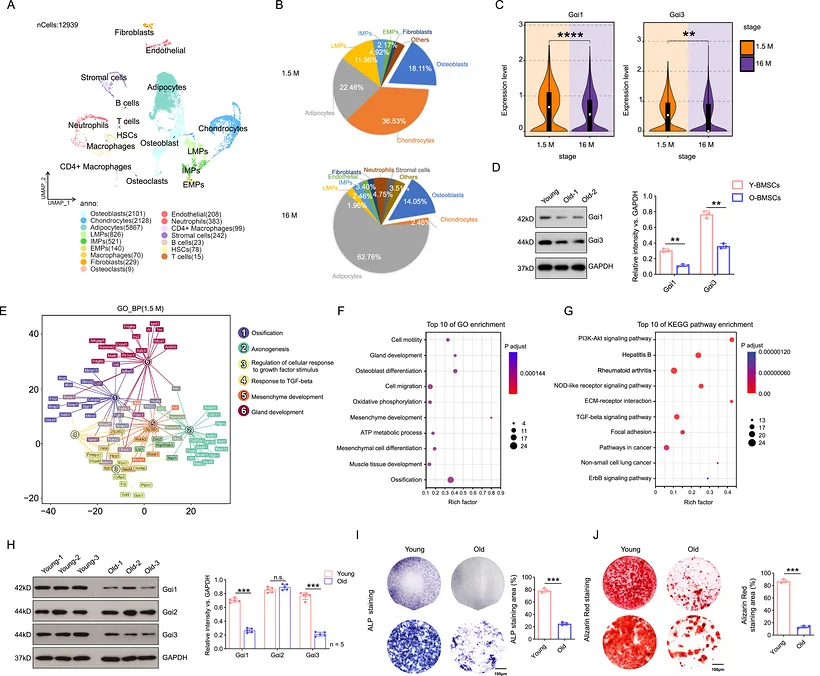
Gαi1 and Gαi3 expression is low in aging osteoblasts and BMSCs, and participate in osteogenic function and signaling. The tSNE plot of visualization of the publicly available bone marrow cells scRNA‐seq data (GEO: #GSE150703) of 1.5‐ and 16‐month‐old mice (A). Proportion of cell types in bone marrow of 1.5 and 16 month‐old mice (B). Expression of Gαi1 and Gαi3 in osteoblasts of 1.5‐ and 16‐month‐old mice (C). Expression of Gαi1 and Gαi3 in BMSCs of young and old mice (D). Biological Processes (BP, E‐F) and KEGG pathways (G) of differentially expressed genes in osteoblasts of 1.5‐ and 16‐month‐old mice. Expression of Gαi subunits in BMSCs of young and old males. Representative images and quantitative analysis of Alp staining and Alizarin red staining of BMSCs from young and old males (H–J). ^∗∗∗^ *p <* 0.001, ^∗∗^ *p <* 0.01, Data were presented as mean ± standard deviation (SD).

We next analyzed differentially expressed genes to identify genes that are co‐expressed with Gαi3 in the osteoblasts of 1.5‐ and 16‐ month‐old mice. Functional enrichment analysis identified the top 10 biological processes (BPs) associated with Gαi3, revealing significant involvement in osteogenesis‐related pathways, including “Ossification”, “Osteoblast differentiation”, “Cell migration,” and others (Figure 1E,F). The KEGG (Kyoto Encyclopedia of Genes and Genomes) enrichment results for Gαi3 CEGs (Co‐Expressing Genes) highlighted 10 key pathways (Figure 1G), including the “TGF‐beta signaling pathway”, “PI3K‐Akt signaling”, with a subset being functionally linked to osteogenic regulation.

BMSCs were isolated from patients with senile osteoporosis and subjected to osteogenic differentiation. The expression of Gαi1/3 in these BMSCs was significantly lower compared to BMSCs from young individuals (Figure 1H). Consistently, ALP and Alizarin red staining demonstrated that the osteogenic capacity of BMSCs from elderly patients was significantly reduced compared to those from young individuals (Figure 1I,J). These results suggest that Gαi1/3 is closely associated with the osteogenic potential of BMSCs.

Gαi1/3 is involved in mouse skeletal development [31]. To determine whether Gαi1 and Gαi3 deficiency can impair bone formation in vivo, we created genetically modified mice with deletion of the Gαi1/3 genes (Gαi1/3 DKO). Newborn Gαi1/3‐DKO pups exhibited reduced body size and growth retardation compared to wild‐type littermates (Figure 2A). We performed whole‐mount skeletal staining of mice 3 days after birth with alcian blue and alizarin red [32]. Specifically, membranous ossification of the skull was impaired, and the clavicles were shorter in Gαi1/3‐DKO newborns than in controls (Figures 2B,C). These findings indicate that Gαi1/3 plays an essential role in skeletal development and bone formation.

**FIGURE 2:**
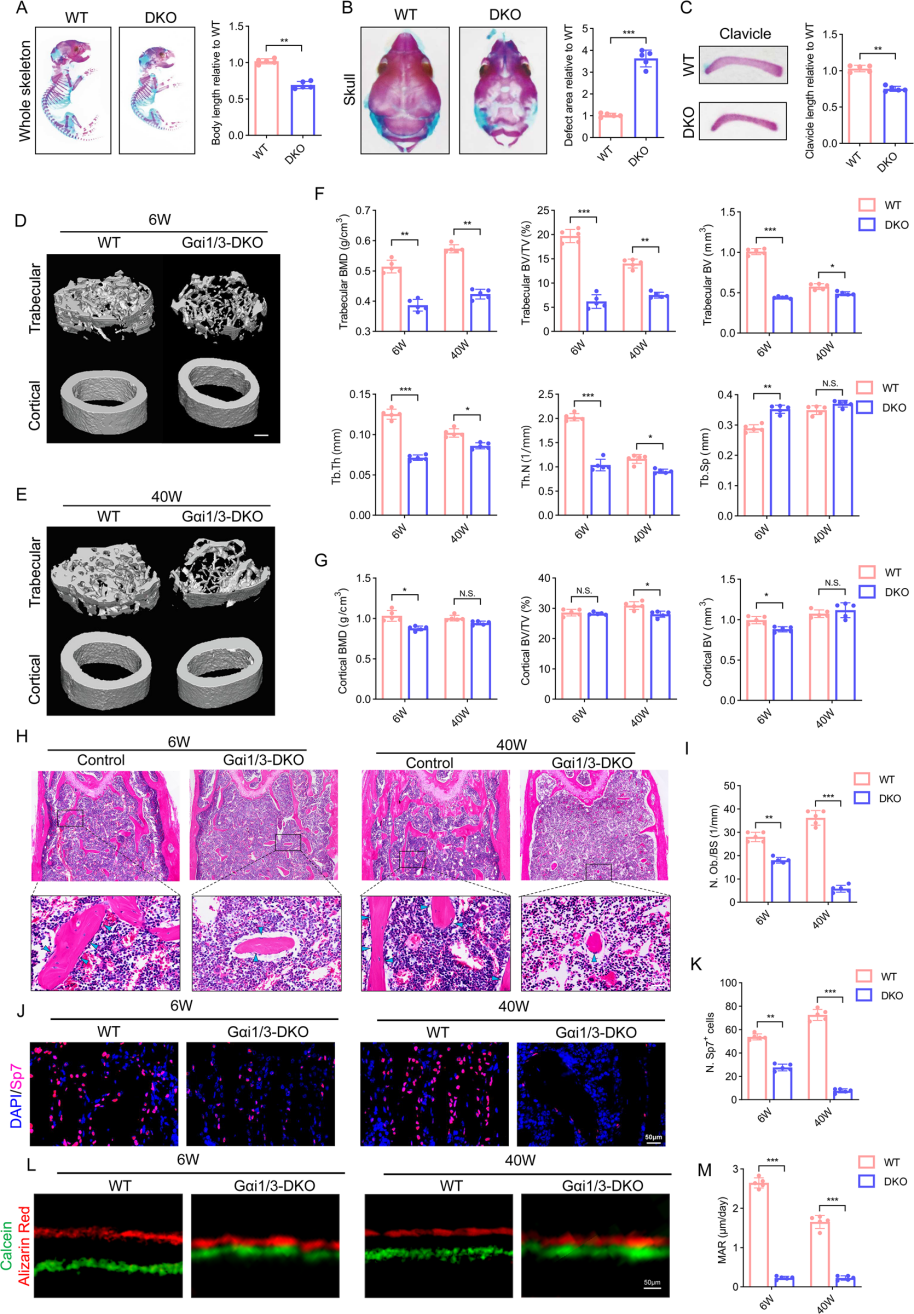
Loss of Gαi1/3 reduces bone formation ability and bone mass. Alcian blue and Alizarin red staining of the whole skeletons, calvarium, and clavicle of the newborn WT and Gαi1/3 DKO mice, and statistical analysis (A–C). µCT analysis of distal femurs from 6‐ and 40‐week‐old WT and Gαi1/3 DKO mice. Representative 3D reconstruction of µCT images (D, E). Quantitative analysis of bone structural parameters in the distal femur (F, G), including BMD, BV, bone volume (BV), bone volume/tissue volume (BV/TV), trabecular thickness (Tb.Th), trabecular number (Tb.N), and trabecular separation (Tb.Sp). Representative images of immunofluorescence (Sp7, Red) staining of femur sections from Gαi1/3‐DKO (J) and quantification of the number of Sp7‐positive cells in bone tissue (K). Calcein labeling to visualize bone formation, with quantification of mineral apposition rate (MAR), Green: Calcein, Red: Alizarin Red (L‐M). ^***^ *p <* 0.001; ^**^ *p <* 0.01; ^*^ *p <* 0.05 versus WT. *n* = 5 per group.

We performed microcomputed tomography (µCT) scans and 3D reconstructions on the femur metaphysis of 6‐week and 40‐week‐old mice. The bone mass of Gαi1/3‐DKO mice was lower than that of wild‐type mice (Figure 2D, E). The µCT analysis revealed decreases in the trabecular bone mineral density (BMD), trabecular bone volume (BV), trabecular bone volume/tissue volume (BV/TV), trabecular numbers (Tb.N), and trabecular thickness (Tb.Th), while trabecular separation (Tb. Sp.) increased significantly in Gαi1/3 knockout mice (Figure 3F). However, the cortical mineral density (BMD), cortical volume (BV), and cortical volume/tissue volume (BV/TV) were not affected in Gαi1/3‐DKO mice (Figure 3G). Taken together, these data suggest that bone mass was reduced in Gαi1/3 knockout mice.

**FIGURE 3:**
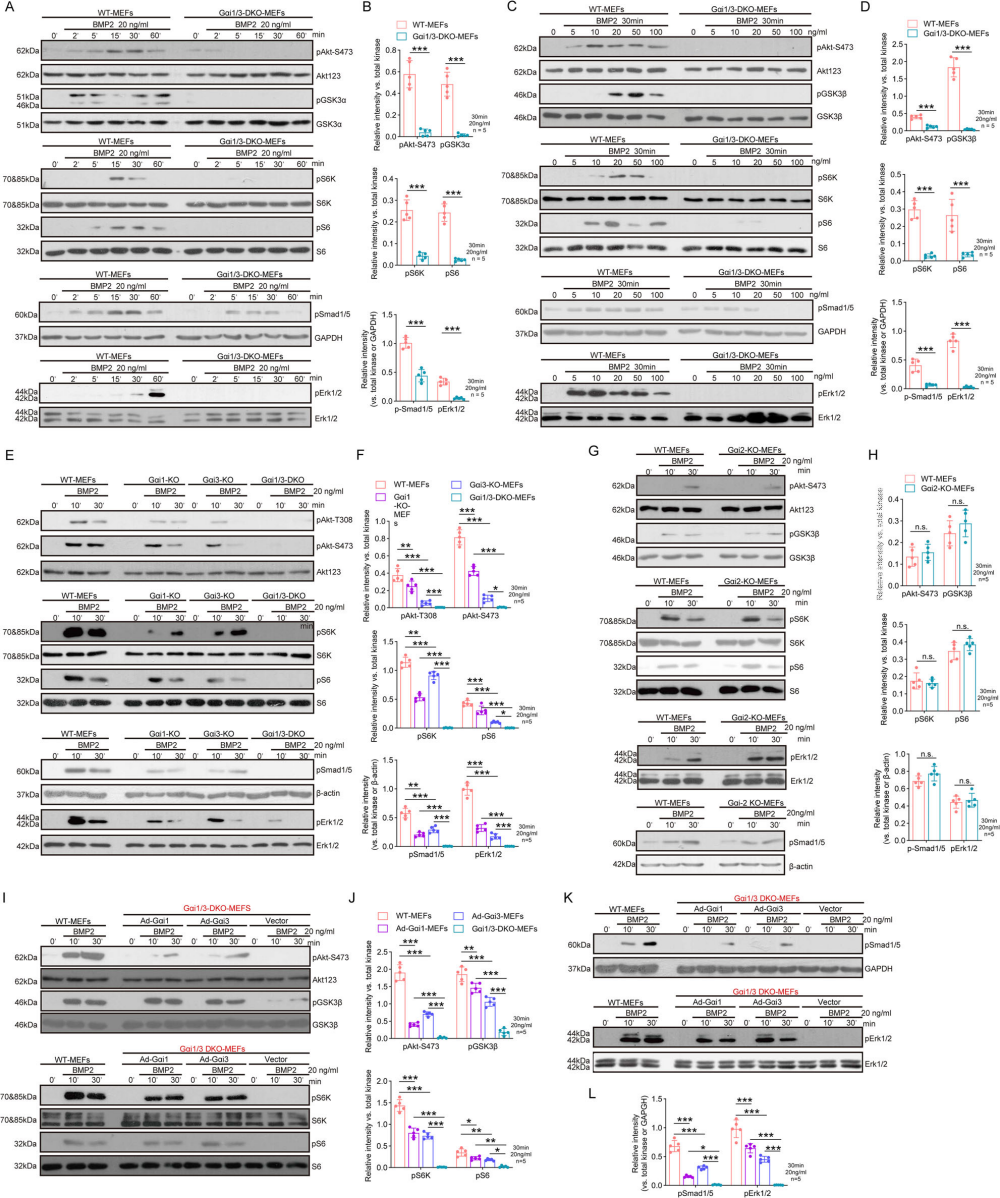
Knockout Gαi1/3 blocked BMP2‐induced activation of Akt‐mTOR and Erk and Smads in MEFs. Wild‐type (WT), Gαi1 and Gαi3 double knockout (Gαi1/3‐DKO) (A–D). Gαi1, Gαi2 or Gαi3 single knockout (SKO) (E–H) mouse embryonic fibroblasts (MEFs) were treated with BMP2 (20 ng/mL) for the applied time, tested by Western blotting of the listed protein. For all Western blotting assays, each lane was loaded with the exact same amount of quantified protein lysates (20 µg in each treatment), the same set of lysate samples was run in parallel (“sister”) gels to test different proteins (Same for all Figures). For the quantification of Western blotting, the indicated proteins were quantified using the ImageJ software (B, D, F, H). Gαi1/3 DKO MEFs were transiently transfected with the adenovirus Gαi1 construct (“Ad‐Gαi1”), the adenovirus Gαi3 construct (“Ad‐Gαi3”) or the empty vector (“Vector”), Different groups were cultured DMEM medium supplemented with BMP2 (20 ng/ml) for indicated time, the proteins listed were examined by Western blotting and phosphorylation (vs total proteins) was quantified (I–L). Quantification was performed from five replicate blot data (*n* = 5, same for the blotting data in all Figures). Data were expressed as mean ± standard deviation (SD, same for all Figures). ^****^ *p* < 0.0001, ^***^ *p* < 0.001, **p < 0.01 vs. WT MEFs.

Consistent with these findings, H&E staining of femurs demonstrated that both the trabecular bone area and the number of osteoblasts on the bone surface (N.ob/B.S) were decreased in Gαi1/3‐DKO mice compared to WT mice (Figure 2H,I). Immunofluorescence staining revealed that the number of Sp7‐positive cells in the bone marrow of Gαi1/3‐DKO mice was significantly lower than that of WT mice (Figure 2J,K). Furthermore, calcein and xylenol orange double‐labeling revealed that Gαi1/3‐DKO mice had a significantly lower cortical bone mineral apposition rate (MAR) compared with WT littermates (Figure 2L,M). Collectively, these results indicate that Gαi1/3‐DKO mice exhibit compromised bone formation and reduced bone mass.

### 3.2 Knockout of Gαi1/3 Blocks BMP2‐Induced Activation of Akt‐mTOR, Erk and Smad Signaling in MEFs

BMP2 is crucial for bone development, maintenance, and regeneration [9, 33]. It promotes osteogenesis by activating downstream signaling pathways, including Akt, Erk, and Smads [34, 35]. Conversely, the inhibition of these pathways suppresses osteogenesis [36, 37]. Our previous studies have shown that Gαi1/3 bind to multiple receptors to mediate downstream signal transduction [16, 17, 38]. We next investigated whether Gαi1/3 interacts with the BMP2 receptor in mouse embryonic fibroblasts (MEFs).

BMP2‐mediated phosphorylation events were compared between WT MEFs and Gαi1/3‐deficient cells [16, 17, 38]. BMP2 treatment (5–100 ng/mL) significantly enhanced phosphorylation of Akt, S6K, Erk1/2 and Smad1/5 in WT MEFs, demonstrating activation of Akt‐mTOR, Erk and Smad pathways (Figure 3A,B). Strikingly, this activation was abolished in Gαi1/3 DKO MEFs, despite comparable total protein levels (Figure 3A,B). These cells were treated with BMP2 (at 5 ng/mL, 10 ng/mL, 20 ng/mL, 50 ng/mL or 100 ng/mL) for 30 min, and the levels of different signaling proteins were examined. In WT MEFs, BMP2 robustly increased the activation of the Akt‐mTOR, Erk‐MAPK, and Smad cascade. However, in Gαi1/3‐DKO MEFs, BMP2‐activated Akt‐mTOR, Erk‐MAPK, and Smad signaling were blocked (Figure 3C,D).

Further studies in MEFs demonstrated that single knockout (SKO) of Gαi1 or Gαi3 resulted in partial inhibition of Akt, S6K, Erk1/2, and Smad1/5 phosphorylation in response to BMP2 (Figure 3E,F), while Gαi1/3 DKO provided almost complete blockage (Figure 3E,F). In MEFs, Gαi3 SKO was more potent than Gαi1 SKO in suppressing BMP2‐induced activation of AKT‐mTOR, Erk, and Smad1/5 (Figure 3E,F). In contrast, BMP2‐induced Akt, S6K, Erk1/2, and Smad1/5 phosphorylation were similar between WT and Gαi2 SKO MEFs (Figure 3G,H). Together, these results show that Gαi1/3 DKO blocks BMP2‐induced AKT‐mTOR, Smad, and Erk activation in MEFs.

To further demonstrate that Gαi1/3 are required for BMP2‐induced signaling, lenti‐CRISPR/Cas9‐Gαi1 KO and lenti‐CRISPR/Cas9‐Gαi3 KO constructs were co‐transfected into WT MEFs, and BMP2‐induced phosphorylation of Akt, Erk1/2, and Smad1/5 was significantly abrogated (Figure 3I,J). Next, we exogenously expressed an adenovirus Gαi1 construct (“Ad‐Gαi1” [18]) or an adenovirus Gαi3 construct (“Ad‐Gαi3” [18]) in Gαi1/3 DKO MEFs, and found that BMP2‐induced phosphorylation of Akt‐S6K, Erk1/2, and Smad1/5 was partially restored in DKO MEFs following ectopic re‐expression of Gαi1 or Gαi3 (Figure 3K,L).

### 3.3 Gαi1/3 Are Required for BMP2‐Induced Signaling in MC3T3‐E1 and BMSCs

To verify the key role of Gαi1/3 in BMP2 signal transduction in osteoblasts, we utilized the small interfering RNA (siRNA) strategy. As described [17, 18], Gαi1 siRNA and Gαi3 siRNA were co‐transfected into MC3T3‐E1 cells. Gαi1 plus Gαi3 double knockdown blocked BMP2‐induced phosphorylation of Akt, S6K, Erk1/2, and Smad1/5 (Figure 4A,B). Significantly, single knockdown of Gαi1 or Gαi3 with siRNA specific for Gαi1 and Gαi3 inhibited BMP2‐induced Akt‐mTOR, Erk, and Smad activation in MC3T3‐E1 (Figure 4A,B). To silence Gαi1/3, Gαi1 and Gαi3 shRNA lentiviral particles were co‐added to MC3T3‐E1 cells, and stable shGαi1/3 MC3T3‐E1 cells were selected. BMP2‐induced Akt, S6K, Smad1/5, and Erk1/2 phosphorylation were almost completely blocked (Figure 4C,D). Total Akt, S6K and Erk1/2 expression were intact (Figure 4C,D). These results demonstrate that Gαi1/3 are required for BMP2‐induced signaling activation in MC3T3‐E1 cells.

**FIGURE 4:**
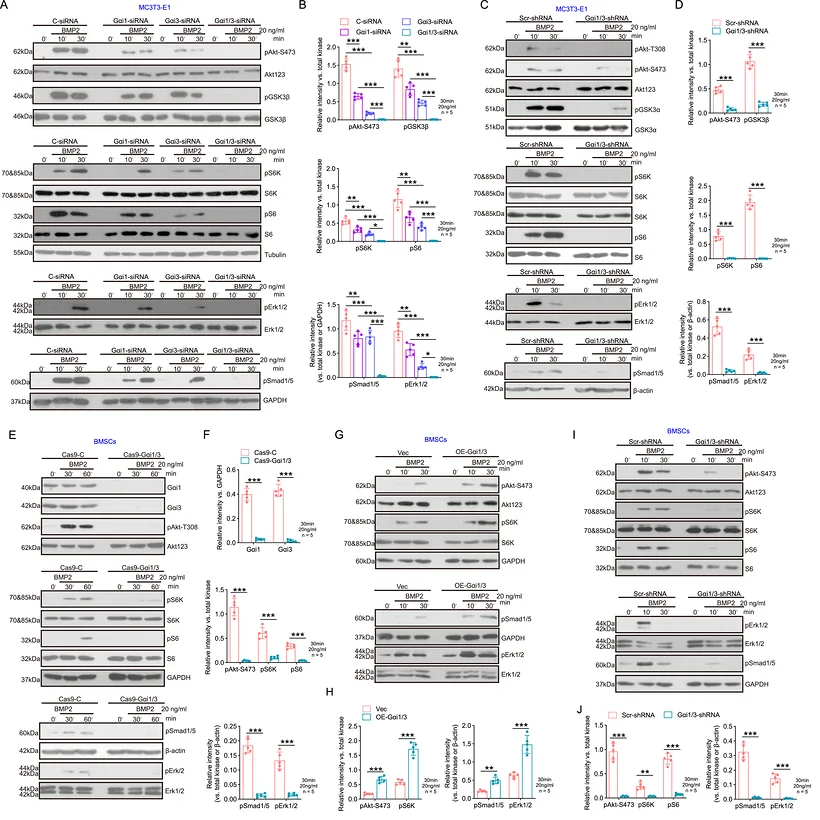
Gαi1/3 are required for BMP2‐induced signaling activation in MC3T3‐E1 and BMSCs. Stable MC3T3‐E1 cells with the scramble control siRNA (“C‐siRNA”), Gαi1 siRNA, and/or Gαi3 siRNA were treated with BMP2 (20 ng/mL) for the applied time and were tested by Western blotting of the listed proteins (A, B). Stable MC3T3‐E1 cells with the scramble control shRNA (“Scr‐shRNA”), Gαi1 shRNA, and Gαi3 shRNA were treated with BMP2 (20 ng/mL) for the applied time and were tested by Western blotting of the listed proteins (C, D). BMSCs isolated from WT mice with the lenti‐CRISPR/Cas9‐Gαi1‐KO construct plus lenti‐CRISPR/Cas9‐Gαi3‐KO construct (“Cas9‐Gαi1/3‐KO”), or the CRISPR/Cas9 control construct (“Cas9‐C”), were treated with BMP2 (20 ng/mL) for the applied time and were tested by Western blotting of the listed proteins (E, F). BMSCs were transfected with the adenovirus Gαi1 construct and the adenovirus Gαi3 construct (OE‐Gαi1/3) or the empty vector. BMSCs in different groups were cultured in DMEM medium supplemented with BMP2 (20 ng/mL), the proteins listed were examined by Western blotting, and phosphorylation was quantified (G, H). BMSCs isolated from WT mice were infected with the lentiviral Gαi1 shRNA and the lentiviral Gαi3 shRNA. Different groups were cultured and treated with BMP2 (20 ng/mL) for 10 or 30 min; the proteins listed were examined by Western blotting, and phosphorylation (vs total proteins) was quantified (I, J). *n* = 5 per group. Data were expressed as mean ± standard deviation (SD, same for all Figures). ^****^ *p* < 0.0001, ^***^ *p* < 0.001, ^**^ *p* < 0.01.

Subsequently, the above results were further verified in BMSCs. To elucidate the functional significance of Gαi1/3 in BMP2 signaling, we performed genetic manipulation in BMSCs through both knockout and overexpression approaches. CRISPR/Cas9‐mediated depletion of Gαi1/3 (“Cas9‐Gαi1/3” BMSCs) significantly attenuated BMP2 (20 ng/mL)‐induced phosphorylation of Akt (Ser‐473), S6K, Smad1/5, and Erk1/2 (Figure 4E,F). Conversely, lentiviral co‐transduction of Gαi1/3 expression vectors (“OE‐Gαi1/3” BMSCs) markedly enhanced these phosphorylation events (Figure 4G,H). Consistent with these findings, shRNA‐mediated knockdown of Gαi1/3 nearly abolished BMP2‐triggered activation of these signaling molecules (Figure 4I,J). These genetic interventions collectively demonstrate that Gαi1/3 are essential positive regulators of BMP2 signaling in BMSCs.

### 3.4 Gαi1 and Gαi3 Are Required for Osteogenic Function Mediated by BMP2

Previous studies have shown that BMP2 activates Akt, MAPK, and Smad to promote osteogenesis of MC3T3‐E1 and BMSCs [34, 39]. The above results show that Gαi1/3 are required for BMP2‐induced Akt‐mTOR, Erk, and Smad1/5 activation in BMSCs and MC3T3‐E1 cells. Based on these findings, we next investigated the effect of Gαi1/3 on BMP2‐mediated osteogenic function.

Examining the effects of Gαi1/3 knockdown in BMSCs (Gαi1/3‐shRNA), EdU staining results suggest that knockdown of Gαi1/3 inhibits the activity of BMSCs and blocks the promoting effect of BMP2 (Figure 5A). Furthermore, the expression of Runx2, Sp7, and OPN proteins was significantly downregulated in the absence of Gαi1 and Gαi3 (Figure 5B). qPCR results were consistent with the above findings, showing that BMP2 could not effectively induce the expression of *Runx2, OPN*, and *Sp7* mRNA in Gαi1/3‐shRNA BMSCs (Figure 5C). In Figures 1 and 2, we confirmed that Gαi1/3 affects BMP2 signal transduction, and Akt‐mTOR, Erk, and Smad1/5 signaling can promote the expression of osteogenesis‐related proteins such as Runx2, Sp7, and OPN. We conclude that the decreased expression of Runx2, Sp7, and OPN in Gαi1/3 deficient BMSCs is mainly caused by the inhibition of osteogenesis‐related signals.

**FIGURE 5:**
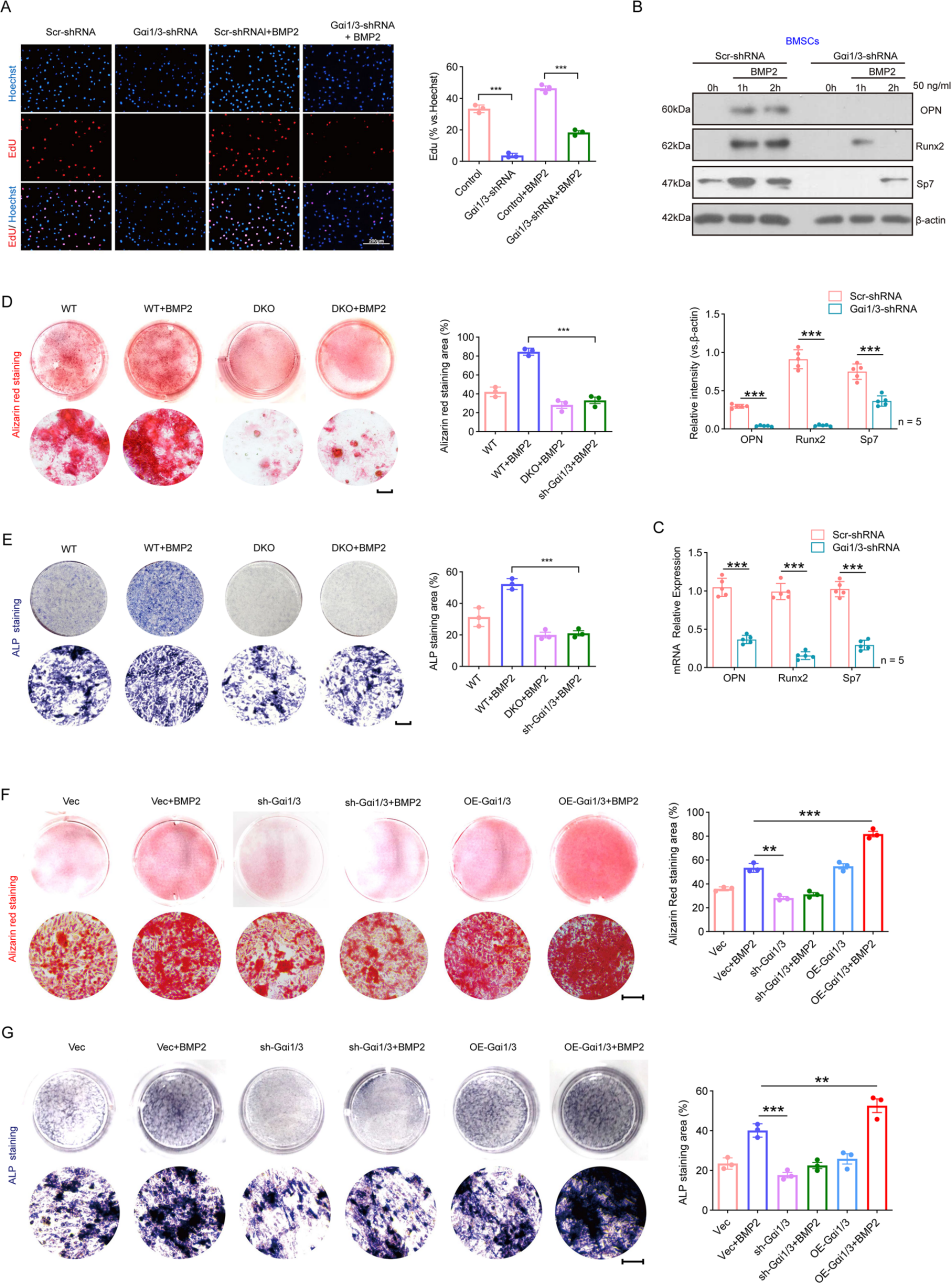
Gαi1 and Gαi3 are required for osteogenic function mediated by BMP2. BMSCs isolated from WT and Gαi1/3 DKO mice were grown in the presence or absence of BMP2 (20 ng/mL). Cell activity (Edu staining) was examined at d5 (A). The expression of Runx2, OPN, and Sp7 was examined by western blotting at different times (B). Runx2, OPN, Sp7, and ALP mRNA were tested by qPCR at 1 h (C). BMSCs from WT and Gαi1/3‐DKO mice. Osteoblast differentiation was induced by medium containing or without BMP2. Mineralization (ALP and Alizarin Red staining) and quantification were measured at d21 (D, E). BMSCs isolated from WT mice were transfected with the lentiviral Gαi1 shRNA and the lentiviral Gαi3 shRNA (sh‐Gαi1/3), or the adenovirus Gαi1 construct and adenovirus Gαi3 construct (OE‐Gαi1/3), and grown in the presence or absence of BMP2 (20 ng/mL). Mineralization (ALP and Alizarin Red staining) and quantification were measured at d21 (F, G). Statistical significance was obtained by Student's t‐tests (two‐tailed). ∗*p <* 0.05; ∗∗*p <* 0.01; ∗∗∗*p <* 0.001. Data are means ± SD.

BMSCs were extracted from WT and Gαi1/3‐DKO mice. Mineralization of BMSCs was measured by ALP and Alizarin Red staining. Compared to WT, results showed that the mineralization of BMSCs from Gαi1/3‐DKO mice decreased. BMP2 was unable to promote the mineralization capacity of BMSCs with Gαi1/3‐DKO (Figure 5D,E). These results indicate that Gαi1 and Gαi3 are required for BMP2‐induced osteogenesis. Collectively, the knockout of Gαi1/3 inhibits BMP2‐induced osteogenesis.

To further verify the above findings, Gαi1/3‐targeting lentiviruses were introduced into BMSCs to achieve knockdown and overexpression of Gαi1/3. Osteogenic differentiation of BMSCs was induced, and the mineralization capacity of Gαi1/3‐DKO was detected by ALP and Alizarin Red staining. The results showed that after knocking down Gαi1/3 (sh‐Gαi1/3), the osteogenic ability of BMSCs decreased significantly. Conversely, overexpression of Gαi1/3 (OE‐Gαi1/3) significantly enhanced the osteogenic ability of BMSCs. Importantly, the osteogenic effect of BMP2 was essentially abolished upon Gαi1/3 knockdown, whereas overexpression of Gαi1/3 significantly potentiated the osteogenic effects of BMP2 (Figures 5F‐G).

BMSCs were extracted from young and old mice. Aged BMSCs were transfected with the lentivirus Gαi1/3 construct (OE‐Gαi1/3). The osteogenic differentiation of BMSCs was assessed by ALP and Alizarin Red staining. Staining and further analysis revealed that, compared to young BMSCs, the osteogenic capacity of old BMSCs was decreased, and overexpression of Gαi1/3 in old BMSCs restored their osteogenic ability (Figure S1A,B). In Gαi1/3‐DKO BMSCs, the osteogenic capacity was decreased, and transfection of the lentivirus Gαi1/3 construct restored the osteogenic ability of BMSCs (Figure S2A,B). These results further confirm that Gαi1 and Gαi3 are required for osteogenic function.

### 3.5 Loss of Gαi1/3 Inhibits the Osteogenesis Induced by BMP2 In Vivo

Following a previously described protocol [20, 21], AAV9‐mRunx2‐Gαi1 shRNA and AAV9‐mRunx2‐Gαi3 shRNA were injected into the femurs of C57B/6 mice. This resulted in the conditional knockdown of Gαi1/3 (Gαi1/3‐CKD), as the viral construct contained the binding sequence for the BMSCs‐specific promoter, Runx2 (Figure S3A–D). After confirming that Gαi1/3 affected BMP2 signal transduction and BMP2‐mediated osteogenesis, an established mouse femoral fracture model was used to compare the healing process of bone fracture between “Gαi1/3‐CKD” and “Vehicle” mice with or without BMP2 treatment. At week 2, the 3D reconstructed images of micro‐CT confirmed that the gap in the fracture sites almost disappeared in the WT BMP2 treatment group, whereas it remained visible in the Gαi1/3‐CKD treatment group (Figure 6A), indicating that BMP2 cannot effectively promote bone regeneration and fracture healing in Gαi1/3‐CKD mice. Microcomputed tomography scan analysis of the femur revealed that the callus mineral bone density (BMD), callus volume (CV), and callus volume/tissue volume (CV/TV) in Gαi1/3‐CKD group after BMP2 treatment were significantly lower compared to the vehicle group (Figure 6B).

**FIGURE 6:**
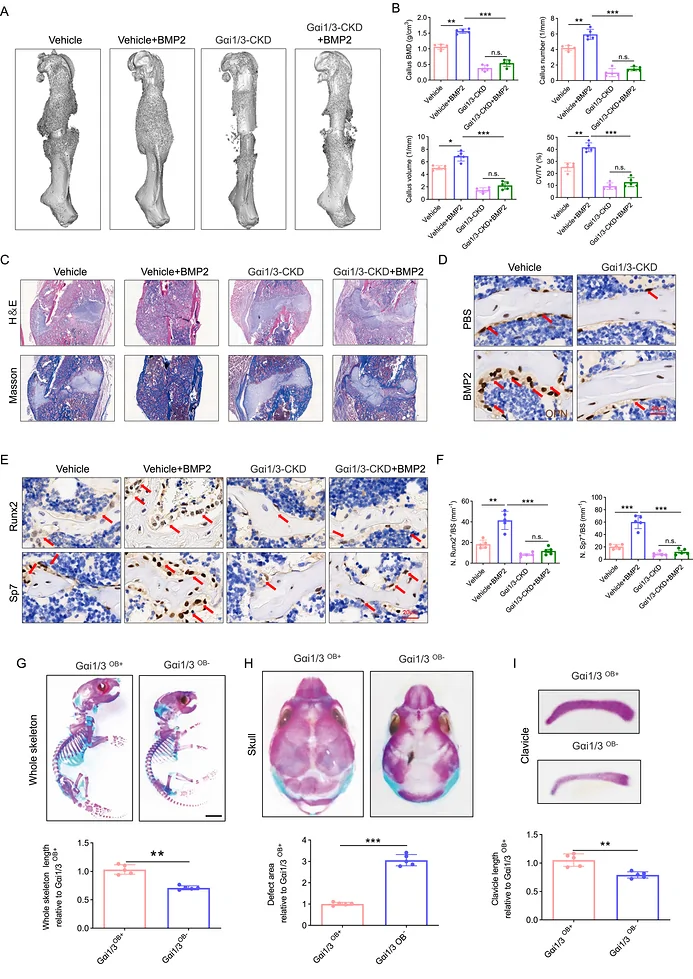
Loss of Gαi1/3 inhibits the osteogenesis induced by BMP2 in vivo. A femoral fracture model was performed in “Gαi1/3‐CKD” and “Vehicle” mice, treated with or without BMP2 treatment. 3D micro‐CT images of the mouse femur fracture zone were taken 2 weeks after surgery, 3D µCT renderings, and quantification of Callus BMD, Callus number, Callus Volume, and CV/TV of different groups (A, B). Representative images of H&E staining of femur fracture sections from mice at week2 post‐surgery (C). Representative images of immunohistochemistry staining and semiquantitative analysis of OPN, Runx2, and Sp7 expression in femur fracture sections from mice at week2 post‐surgery (D–F). Alcian blue and Alizarin red staining of the whole skeletons, calvarium and clavicle of the newborn Gαi^OB+^ mice and Gαi^OB−^ mice, and statistical analysis (G–I). Statistical significance was obtained by Student's *t*‐tests (two‐tailed). ∗*p <* 0.05; ∗∗*p <* 0.01; ∗∗∗*p <* 0.001. Data are means ± SD.

H&E and Masson staining were performed to evaluate the histological properties of newly mineralized bone tissues. The fracture site of the Gαi1/3‐CKD + BMP2 group was covered by fewer calluses, and the bone remodeling was slower when compared with the WT group treated with BMP2 at week 2 post‐surgery (Figure 6C). Moreover, immunohistochemistry (IHC) staining and semiquantitative scoring revealed decreased OPN, Runx2, and Sp7 expression observed in the Gαi1/3‐CKD group. Thus, BMP2 cannot promote OPN, Runx2, or Sp7 expression in vivo (Figure 6D‐F), suggesting that Gαi1/3 exerts beneficial effects on BMP2‐mediated bone formation and remodeling.

To further investigate the physiological role of Gαi1/3 in the commitment of BMSCs to the osteoblast lineage, we generated Col1a1‐cre; Gαi^fl/fl^ mice (hereafter referred to as Gαi^OB−^ mice) by crossing Gαi^fl/fl^ mice with Col1a1‐Cre (Gαi^OB+^) mice (Figure S3C,D). Gαi was knocked out in BMSC‐derived osteoblasts Gαi^OB−^ mice. Newborn Gαi^OB−^ mice exhibited a significantly smaller body size than their control littermates (Figure 6G). Moreover, a delay in bone formation was observed in the skull and clavicles of newborn Gαi^OB−^ mice (Figure 6H‐I). Notably, Gαi^OB−^ mice had difficulty surviving for more than one week. These results indicate that Gαi1/3 plays an important role in BMP2‐mediated bone formation.

### 3.6 The Adaptor Protein Gab1, Downstream of Gαi1/3, Mediates BMP2‐Induced Signaling Activation

Following RTK stimulation, Gab1 translocates to the plasma membrane where it undergoes phosphorylation, subsequently functioning as a scaffold protein to facilitate downstream signaling propagation [40]. Consistent with this mechanism, our findings reveal that EGFR activation induces Gab1‐Gαi complex formation, facilitating signal transmission from the EGFR‐Gαi axis to the p85 regulatory subunit [16]. BMP receptors represent a unique class of human transmembrane proteins possessing intrinsic serine/threonine kinase activity [14]. Inhibition of BMPRI/II endocytosis can inhibit BMP2‐induced signaling and osteogenesis [41]. Ligand‐binding events induce the transcription of multiple osteogenesis‐associated genes through the activation of a complex array of downstream intracellular mediators that includes PI3K‐Akt‐mTORC1, Erk‐MAPK, and the Smads pathway [13, 14, 42]. Previously, our research group confirmed that Gαi1/3 and Gab1 are coupled to mediate the activation of downstream signal pathways following stimulation with BDNF and VEGF [17, 18]. However, the role of Gab1 in serine/threonine kinase receptor signal transduction remains unclear. We therefore tested whether Gαi1/3 mediates BMP2 downstream signal transduction through the coupling and activation of Gab1.

In western blotting studies, we found that Gαi1/3 DKO blocked BMP2‐induced Gab1 activation (Tyr‐627 phosphorylation, Figure 7A,B), whereas Gαi2 SKO had no significant effect on Gab1 activation (Figure 7C). In WT MEFs, Gab1 activation by BMP2 was significantly attenuated by the knockout of Gαi1 and/or Gαi3 (Figure 7D). In Gαi1/3 DKO MEFs, re‐expression of Gαi1 or Gαi3 partially restored BMP2‐induced Gab1 activation (Figure 7E). In BMSCs, overexpression of Gαi1/3 also promoted BMP2‐induced Gab1 activation (Figure 7F). Conversely, in Gαi1/3‐shRNA BMSCs, the ability of BMP2 to induce Gab1 phosphorylation was significantly reduced (Figure 7G). These results show that Gαi1/3 are essential for BMP2‐induced Gab1 activation, a necessary step for downstream Akt, Erk1/2, and Smad1/5 activation of serine/threonine kinase receptors.

**FIGURE 7:**
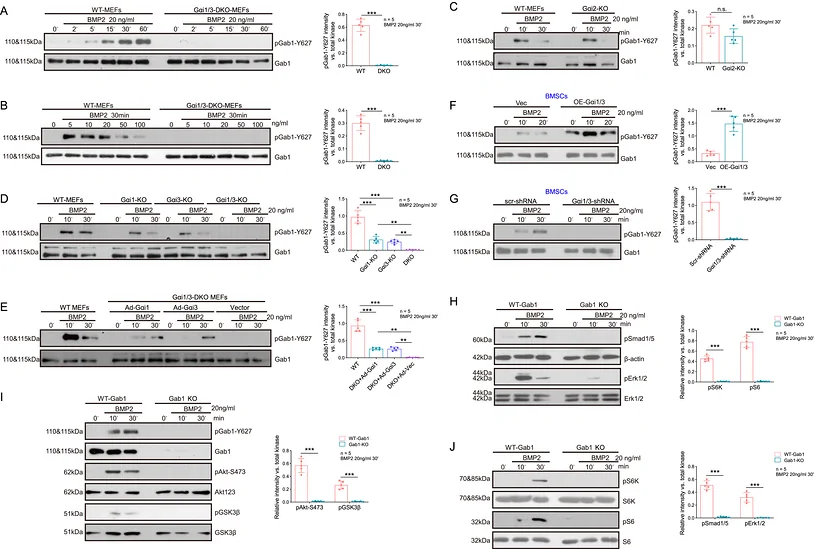
The adaptor protein Gab1, downstream of Gαi1/3, mediates BMP2‐induced signaling activation. WT, Gαi1/3 DKO MEFs, and Gαi1, Gαi2, Gαi1 KO MEFs were treated with BMP2 (20 ng/mL) for the applied time period; p‐Gab1 and Gab1 were shown (A–D). Gαi1/3 DKO MEFs were transiently transfected with the adenovirus Gαi1 construct (“Ad‐Gαi1”), the adenovirus Gαi3 construct (“Ad‐Gαi3”), or the empty vector (“Vec”), BMP2‐induced Gab1 activation was tested (E). BMSCs were transiently transfected with the adenovirus Gαi1 and Gαi3 constructs (“OE‐Gαi1/3”), BMP2‐induced Gab1 activation was tested (F). BMSCs were transfected with the lentiviral Gαi1 shRNA and the lentiviral Gαi3 shRNA (Gαi1/3‐shRNA), BMP2‐induced Gab1 activation was tested (G). WT MEFs or Gab1 knockout MEFs were treated with BMP2 (20 ng/mL), BMP2 (20 ng/mL)‐activated signaling was tested, and protein phosphorylation was quantified (H‐J). Quantifications were from five biological replicates. Statistical significance was obtained by Student's t‐tests (two‐tailed). ∗*p <* 0.05; ∗∗*p <* 0.001; ∗∗∗*p <* 0.001. Data are means ± SD.

In Gab1 KO BMSCs, BMP2‐induced phosphorylation of Akt, S6K, Erk1/2, and Smad1/5 was significantly blocked (Figure 7H‐J). Thus, Gab1 plays an essential role in the activation of BMP2‐induced signaling mediated by Gαi1/3. Furthermore, Gab1 serves as a critical adaptor protein for the downstream signal activation of serine/threonine kinase receptors.

Gαi1/3 DKO BMSCs were transiently transfected with the adenovirus Gab1 construct (“Ad‐Gab1”), the adenovirus Gαi3 construct (“Ad‐Gαi3”) or the empty vector (“Vector”). Overexpression of Gab1 in Gαi1/3 DKO BMSCs did not restore the transduction of downstream signals by BMP2. Western blot results showed that the phosphorylation of Akt, S6K, Erk1/2, and Smad1/5 was significantly inhibited in Gαi1/3 DKO BMSCs. Overexpression of Gab1 (Ad‐Gab1) in Gαi1/3 DKO BMSCs did not affect the phosphorylation of downstream proteins (Figure S4A). Furthermore, upon inducing osteogenic differentiation in these cells, ALP and Alizarin Red staining revealed that overexpressing Gab1 in Gαi1/3 DKO BMSCs did not restore their osteogenic capacity (Figure S4B–D). These results demonstrate that Gαi1/3 mediates downstream signal transduction and osteogenesis via Gab1.

### 3.7 Mutants of Gαi1 and Gαi3 Disrupt BMPR1A/B Binding of Adaptor Proteins and Prevent Activation of Downstream Signaling

Protein‐protein interaction analysis performed using PyMOL identified and categorized functional residues based on their binding interfaces. Specifically, hydrogen bond interactions were observed between Gαi3 and BMPR1A/B, as well as between Gαi3 and Gab1 (Figure 8A–C). Similarly, multiple residue clusters were identified at the interfaces of Gαi1 and BMPR1A, BMPR1B, and Gab1 (Figure S5A–C). Collectively, these interactions suggest that Gαi1/3 may facilitate the binding of BMPR1A/B to Gab1, thereby activating downstream signaling

**FIGURE 8:**
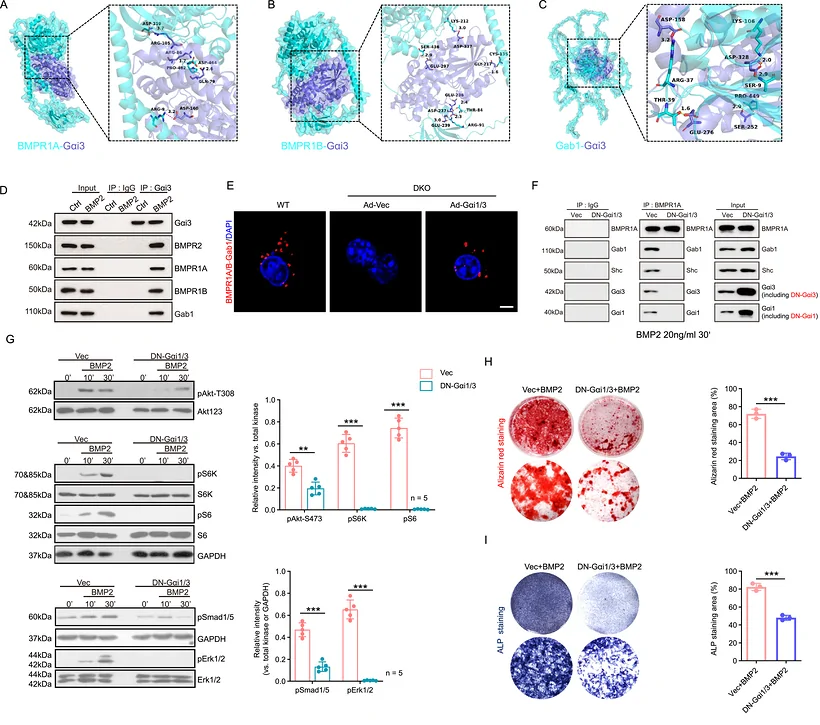
Mutants of Gαi1 and Gαi3 disrupt BMPR1A/B binding of adaptor proteins and prevent activation of downstream signaling. The Gαi3 protein is represented as a slate cartoon model, BMR1A/BMR1B/Gab1 protein is shown as a cyan cartoon model, and their binding sites are shown as the corresponding‐colored stick structure. When focusing on the binding region, the binding site is then shown as a presentation of the protein to which it belongs (A–C). BMSCs were treated with BMP2 (20 ng/mL) for 30 min; BMPRs‐Gαi1/3‐Gab1 association (“IP”) and expression (“Input”) were shown (D). BMSCs isolated from WT and DKO mice were treated with BMP2 (20 ng/mL) for 30 min; in situ PLA technology indicated endogenous BMPR1A/B‐Gab1 protein interactions in BMSCs. Red, positive interaction complexes (D). Scale bar: 10 µm. Stable WT BMSCs, with the DN mutant‐Gαi1 (murine) construct plus the DN‐Gαi3 construct (“DN‐Gαi1/3”) or the vector control (“Vec”), were treated with BMP2 (20 ng/mL) for 30 min. The association of BMR1A, Grb1, Shc, Gαi1, and Gαi3 was examined by co‐immunoprecipitation (Co‐IP) assays (F), and their expressions are shown in “Input” (F). The listed proteins in membrane fraction lysates and total cell lysates were examined (G). The osteogenic ability of BMSCs (“DN‐Gαi1/3”, “Vec”) was detected by ALP and Alizarin Red staining at d21 (H‐I). Statistical significance was obtained by Student's *t*‐tests (two‐tailed). ^∗^ *p <* 0.05; ^∗∗^ *p <* 0.01; ^∗∗∗^ *p <* 0.001. Data are means ± SD.

Co‐immunoprecipitation (Co‐IP) results showed that following BMP2 stimulation, Gαi1/3 associated with BMP2 receptors(BMPR2, BMPR1A/B)and Gab1 (Figure 8D; Figure S5D). Consistently, in situ proximity ligation assay (PLA) confirmed that the direct interaction between endogenous BMPR1A/B and Gab1 was abolished in Gαi1/3‐DKO BMSCs (Figure 8E). Introducing Gαi1/3 into Gαi1/3‐DKO BMSCs restored the mutual binding of BMPR1A/B and Gab1 (Figure 8E, Red). The above results confirm that Gαi1/3 binds to BMP2 receptors and Gab1, mediating downstream signal transduction.

To investigate Gαi1/3‐dependent BMP2 signaling mechanisms, we employed dominant‐negative (DN) constructs, in which the conserved Gly (G) residue in the G3 box was substituted with Thr (T) to disrupt Gαi1/3‐protein interactions [43]. The DN‐Gαi1 and DN‐Gαi3 constructs were co‐transduced into WT BMSCs, and after selection, stable BMSCs, “DN‐Gαi1/3”, formed. As BMPR1A binds to BMP2 with high affinity and is the main receptor for BMP2 signaling [44, 45], we focused on the impact of Gαi1/3 mutation on BMPR1A. Co‐IP assay results shown in Figure 8A demonstrate that DN‐Gαi1/3 disrupts BMP2 While BMP2 treatment induced BMPR1A‐Gab1 binding (without affecting expression, Figure 8E Input), this interaction was dependent on functional Gαi1/3 (Figure 8F). Western analysis verified DN mutant expression (Figure 8E Input). Importantly, DN‐Gαi1/3 expression markedly suppressed BMP2‐triggered Smad1/5, Akt, and Erk1/2 phosphorylation (Figure 8G). This indicates that Gαi1/3 dominant negative mutants disrupt BMP2‐induced BMP2 receptor association with Gab1 and BMP2 signaling activation. The results of ALP and Alizarin Red staining showed that the mutation of Gαi1/3 inhibited BMP2‐induced osteogenesis of BMSCs (Figure 8H,I). In conclusion, Gαi1 and Gαi3 mutants disrupt BMPR1A/B binding of adaptor proteins and prevent activation of downstream signaling.

Our findings establish the Gαi1/3–Gab1 signaling axis as a critical and novel component of the BMP2 receptor (serine/threonine kinase) signal transduction pathway, which is essential for BMP2‐induced osteogenesis both in vitro and in vivo. This work expands the functional repertoire of Gαi protein beyond GPCRs and RTKs, and identifies Gαi1/3 as potential therapeutic targets for enhancing bone formation and treating bone disorders such as fractures and osteoporosis (Figure S6).

## 4 Discussion

A previous study has confirmed that penetrance and expressivity of the rib fusion phenotype are altered in mice knockout Gαi [29]. However, the molecular mechanism of Gαi1/3 in bone development and bone formation is not clear. Our research highlights the pivotal roles of Gαi1 and Gαi3, key members of the Gαi protein family, in the process of bone formation. We found that Gαi1/3 expression was abnormally decreased in senile osteoporosis. Our results corroborate the findings of Birnbaumer by demonstrating delayed skeletal development in newborn Gαi1/3‐DKO mice [29]. In addition, bone mass and quality decreased in Gαi1/3‐DKO mice. These results suggested that Gαi1/3 may be involved in bone formation and development.

Previous studies have found that the Gα(i) subfamily of heterotrimeric G proteins regulates the development of skeletons [29]. Birnbaumer et al. proposed that Gαi may affect signals such as BMPs, Wnt, Hedgehog, etc., which are closely related to bone development. Gαi3 knockdown significantly decreased the osteogenic differentiation ability of stem cells of dental apical papilla (SCAPs) [46]. Based on the above research, this study explored the expression of Gαi in BMSCs and osteoblasts. With aging, both in mice and humans, the osteogenic capacity of bone marrow mesenchymal stem cells (BMSCs) or osteoblasts declines, which is accompanied by reduced expression of Gαi1/3. In contrast, no significant change was observed in the expression of Gαi2.

In Gαi1/3‐DKO mice, the skeletal development of neonatal mice was delayed. The bone mass of adult mice was lower than that of control mice, and their bone formation ability was insufficient. Although Gαi2 shows no change in senile osteoporosis, its influence on bone metabolism cannot be ruled out, and the role of Gαi2 in bone metabolism is still worthy of further exploration.

In the physiological process of bone formation and regeneration, expression of BMP2 and its binding to the BMPRI/II receptor leads to activation of multiple downstream signal pathways including ERK, PI3K‐Akt‐mTOR, and Smads [14, 47]. Activation of these cascades promotes BMSCs and Osteogenic precursor cells proliferation, differentiation, and osteogenesis [14]. Previous studies have demonstrated that impaired signal transduction in response to BMP‐2 underlies delayed fracture healing and osteoporosis [48]. Recombinant human bone morphogenetic protein‐2 (hrBMP‐2) has been used clinically to treat delayed fracture healing [49]. However, the underlying mechanisms of BMP2‐induced activation of Akt, Erk, and Smads remain largely unknown.

The present study provides a novel mechanism for the role of Gαi1/3 in BMP2 signal transduction and osteogenesis. We show that Gαi1/3 are essential players in BMP2‐induced Akt, Erk, and Smad signaling activation. In BMSCs, MC3T3‐E1 or MEFs, Gαi1/3 KO/shRNA, CRISPR‐induced Gαi1/3 gene editing, or dominant negative Gαi1/3 mutations potently inhibit BMP2‐induced downstream activation of Akt, Erk, and Smads. Overexpression of Gαi1/3 facilitated Akt activation by BMP2. Functional studies demonstrate that MC3T3‐E1/BMSCs with Gαi1/3 deficiency were refractory to osteogenesis. Thus, Gαi1/3 are required for BMP2‐induced downstream activation of Akt, Erk, and Smads, which then lead to osteogenesis.

Furthermore, we constructed Gαi1/3 knockdown mice (Gαi1/3‐CKD) in BMSCs. In Gαi1/3‐CKD mice, fracture healing is delayed, and the bone healing‐promoting effect of BMP2 disappears. Although we failed to construct a fracture model in Gαi1/3 conditional knockout mice (Gαi1/3^BMSC−^ mice), we confirmed that bone development was delayed in Gαi1/3^BMSC−^ mice. The above results confirm that Gαi1/3 plays an indispensable role in bone formation, and its deficiency inhibits the osteogenic effect of BMP2.

Our findings expand on the BMP signaling mechanism and confirm that Gαi1/3 mediates the signal transduction of serine/threonine kinase type receptors (BMPRI/II). Gab1 functions as a critical scaffolding adaptor that mediates essential signaling transduction pathways [50]. Accumulated evidence indicates that Gab1 proteins can function to activate SHP2 phosphatase, which in turn activates MAPK signaling [51]. In addition, the Gab1–p85 interaction is important in activating the PI3K/Akt pathway in mammalian cells [52, 53]. Activation of Akt and Erk and osteoblast function were impaired in Gab1‐deficient osteoblasts [50].

Prior work from our group has established that Gαi1/3 mediate Gab1 recruitment and activation of multiple growth factors in RTK signaling pathways [16, 38]. In the present study, we show that Gαi1/3 are essential for Gab1 activation in response to BMP2. In Gab1 KO BMSCs, BMP2‐induced Akt, Erk, and Smad activation was completely blocked. Therefore, Gαi1/3 are required for BMP2‐induced recruitment and activation of Gab1, facilitation of PI3K‐Akt and MAPK, and activation and osteogenesis. Without functional Gαi1/3, Gab1 cannot be recruited and activated in response to BMP2, resulting in defective downstream signaling and osteogenesis. We speculate that Gαi1/3 may also be involved in BMPRI/II (the BMP2 receptor). Subsequent studies have also confirmed that Gαi1/3 can bind to BMPRI/II‐Gab1. The detailed mechanisms may require further characterization.

In this study, we found aberrant expression of Gαi1/3 in the bone tissue of patients with osteoporosis. In Gαi1/3 DKO mice, the bone mass and quality decreased. We confirmed BMP2 as an important cytokine to promote osteogenesis, while signal transduction and osteogenesis are dependent on Gαi1/3. Loss of Gαi1/3 caused delayed fracture healing. In addition to BMP2 signaling, Hedgehog and Wnt signaling also participate in bone development, maintenance, and regeneration [54]. Wnt3a signaling to β‐catenin is dependent on heterotrimeric G proteins, as we found that Gαi1/3 mediates Wnt/β‐catenin signal transduction [55]. The Drosophila Hedgehog signaling cascade has been shown to engage G protein signaling through Smoothened‐mediated mechanisms [56]. Suppression of Gnai2 and Gnai3 expression attenuates Shh‐mediated proliferation in rat cerebellar granule cell precursors [57]. These studies suggest that Gαi1/3 may regulate the signal pathways activated by multiple cytokines to affect osteogenesis.

While Gαi1/3 plays a role in osteoclasts, the current study establishes its novel and independent role as a critical transducer of BMP2 signaling in the osteoblast lineage. Interestingly, while our previous work identified Gαi1/3 as a mediator of RANKL‐induced osteoclastogenesis [58], the current findings demonstrate that it is also essential for BMP2‐mediated osteogenesis. This suggests that Gαi1/3 acts as a molecular switch in the bone microenvironment, ensuring coupled remodeling by responding to both resorptive and formative stimuli.

## 5 Conclusions

This study establishes the Gαi1/3‐Gab1 axis as a novel and essential signaling module for BMP2‐induced osteogenesis. We demonstrate that Gαi1/3 are indispensable for BMP2 receptor (BMPRI/II) complex formation with the adaptor protein Gab1, which is required for the subsequent activation of downstream PI3K‐Akt‐mTOR, Erk, and Smad pathways both in vitro and in vivo. Genetic ablation of Gαi1/3 in mice leads to skeletal defects, reduced bone mass, and impaired BMP2‐mediated fracture healing. Mechanistically, Gαi1/3 act as critical scaffolds bridging BMP2 receptors to Gab1, facilitating signal transduction. These findings expand the functional repertoire of Gαi proteins beyond GPCRs and RTKs to include receptor serine/threonine kinases. Consequently, targeting the Gαi1/3‐Gab1 axis presents a promising therapeutic strategy for enhancing bone formation and treating conditions such as fractures and osteoporosis.

## Author Contributions

**Chaowen Bai**: investigation, formal analysis, software, resources. **Yifei Zhu**: supervision. **Yitian Yang**: software. **Jinyu Bai**: supervision, resources. **Chang She**: funding acquisition. **Qize Sun**: software. **Cong Cao**: investigation, visualization, validation, project administration, software. **Huajian Shan**: investigation, funding acquisition, writing – original draft, writing – review and editing, formal analysis, data curation, conceptualization, visualization, supervision. **Sonngtao Li**: funding acquisition. **Xiaozhong Zhou**: funding acquisition, conceptualization. **Chenyang Xu**: formal analysis.

## Conflicts of Interest

The authors declare no conflicts of interest.

## Supporting information




**Supporting File 1**: advs76837‐sup‐0001‐SuppMat.docx.

**Supporting File 2**: advs76837‐sup‐0002‐Figure S1‐S6.pdf.

## Data Availability

The data that support the findings of this study are available in the supplementary material of this article.
