# Supplementary material for: Gαi1/3 Are Essential for BMP2‐Induced Osteogenesis by Bridging BMP Receptors to the Gab1 Signaling Complex
Source: Adv Sci (Weinh). 2026 Aug 10;13(59):e76837. doi: 10.1002/advs.76837 (PMC13454971; doi:10.1002/advs.76837)
Supplement: Supplementary file 1 [file ADVS-13-e76837-s002.pdf]

Figure S1

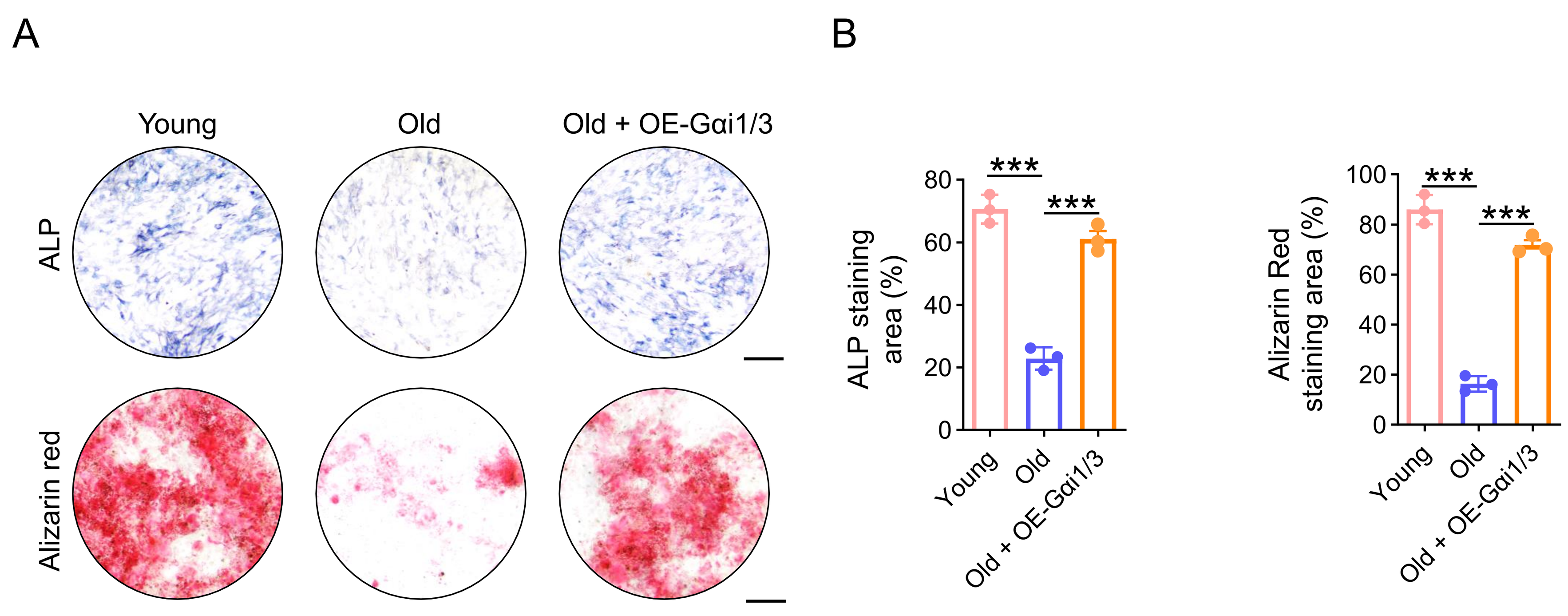

Figure S2

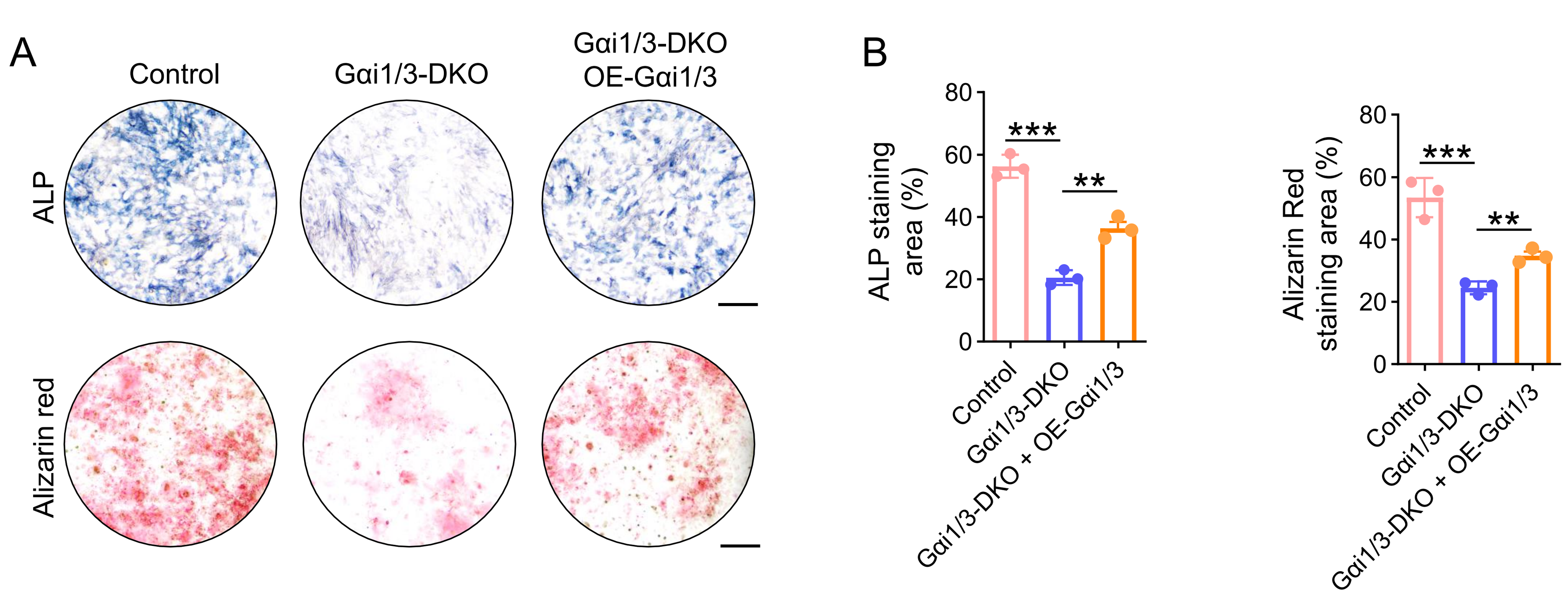

Figure S3

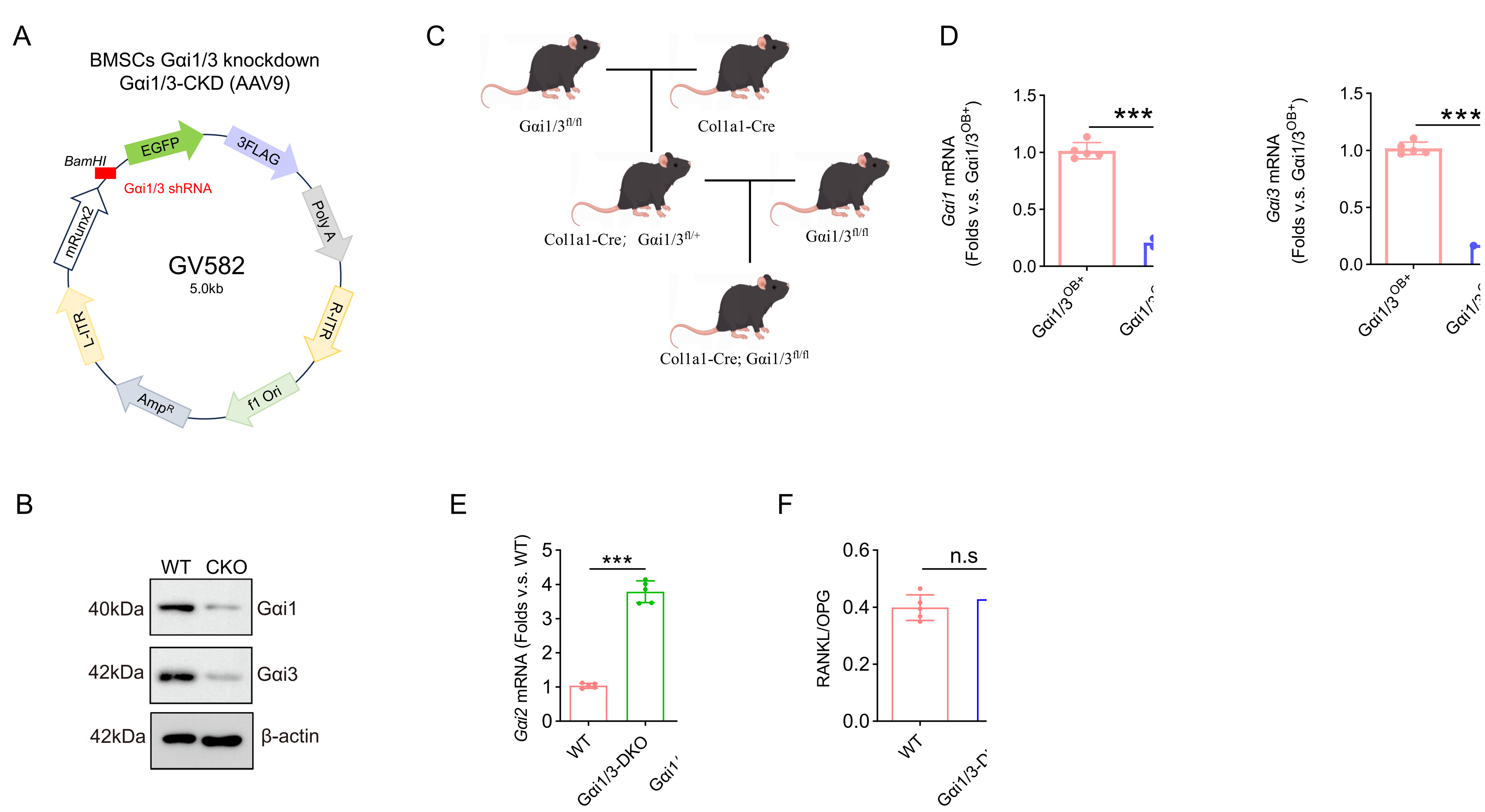

Figure S4

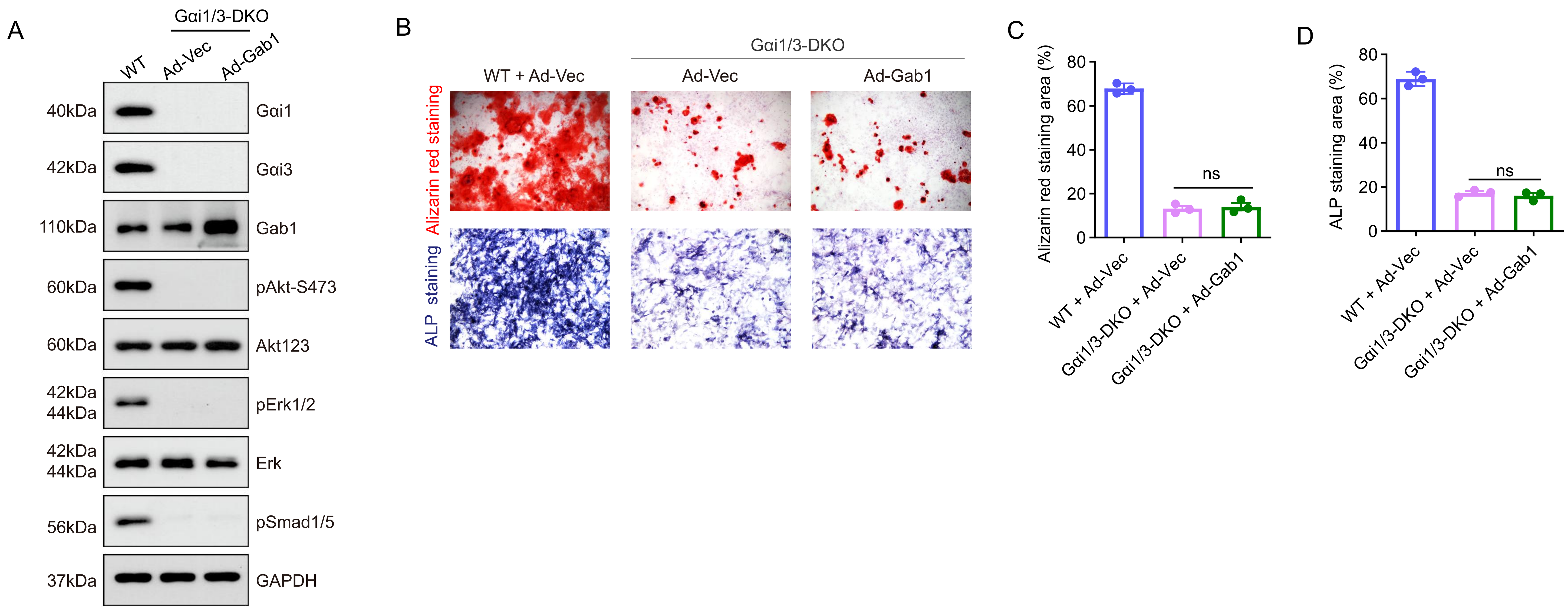

Figure S5

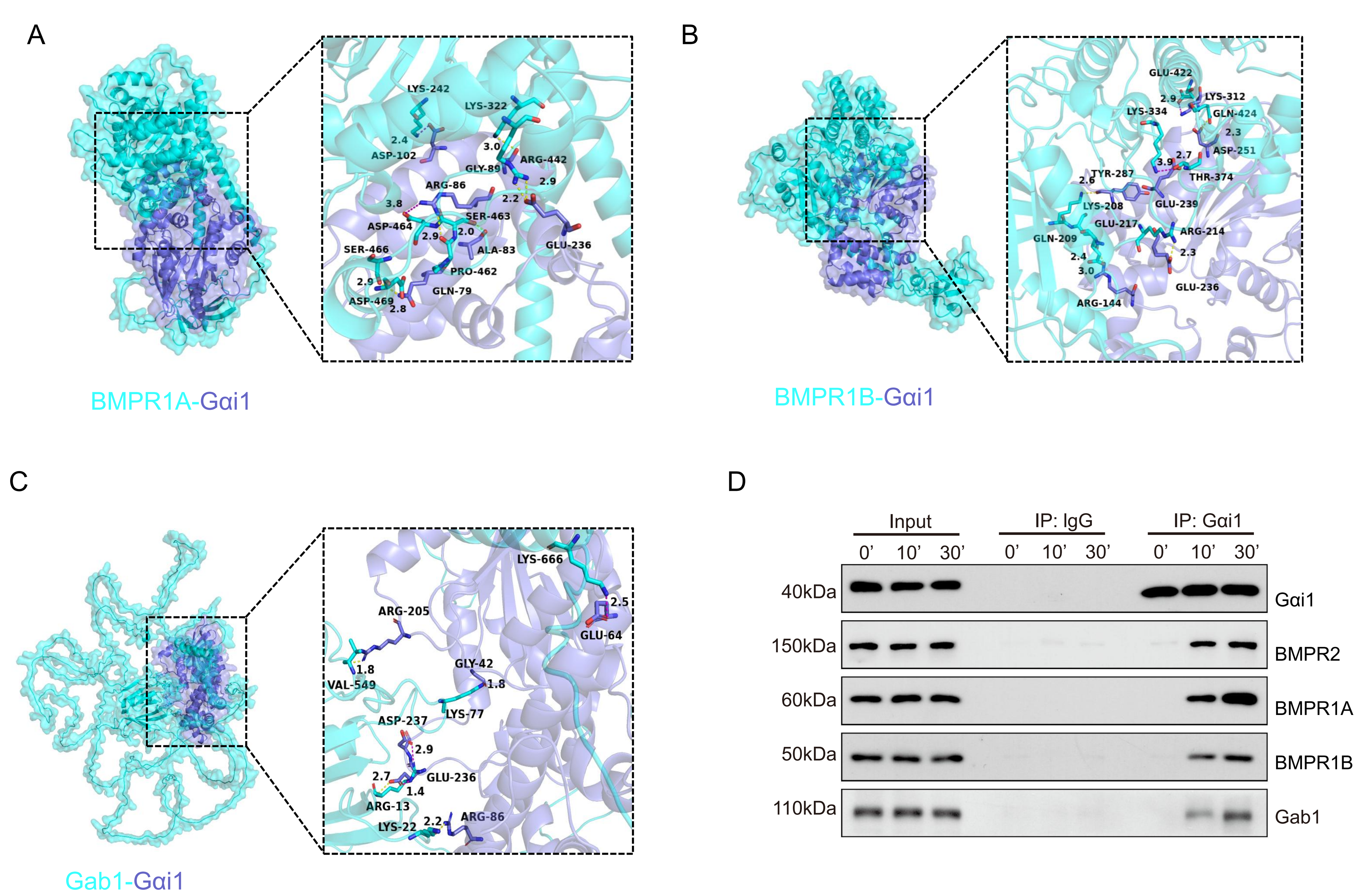

Figure S6

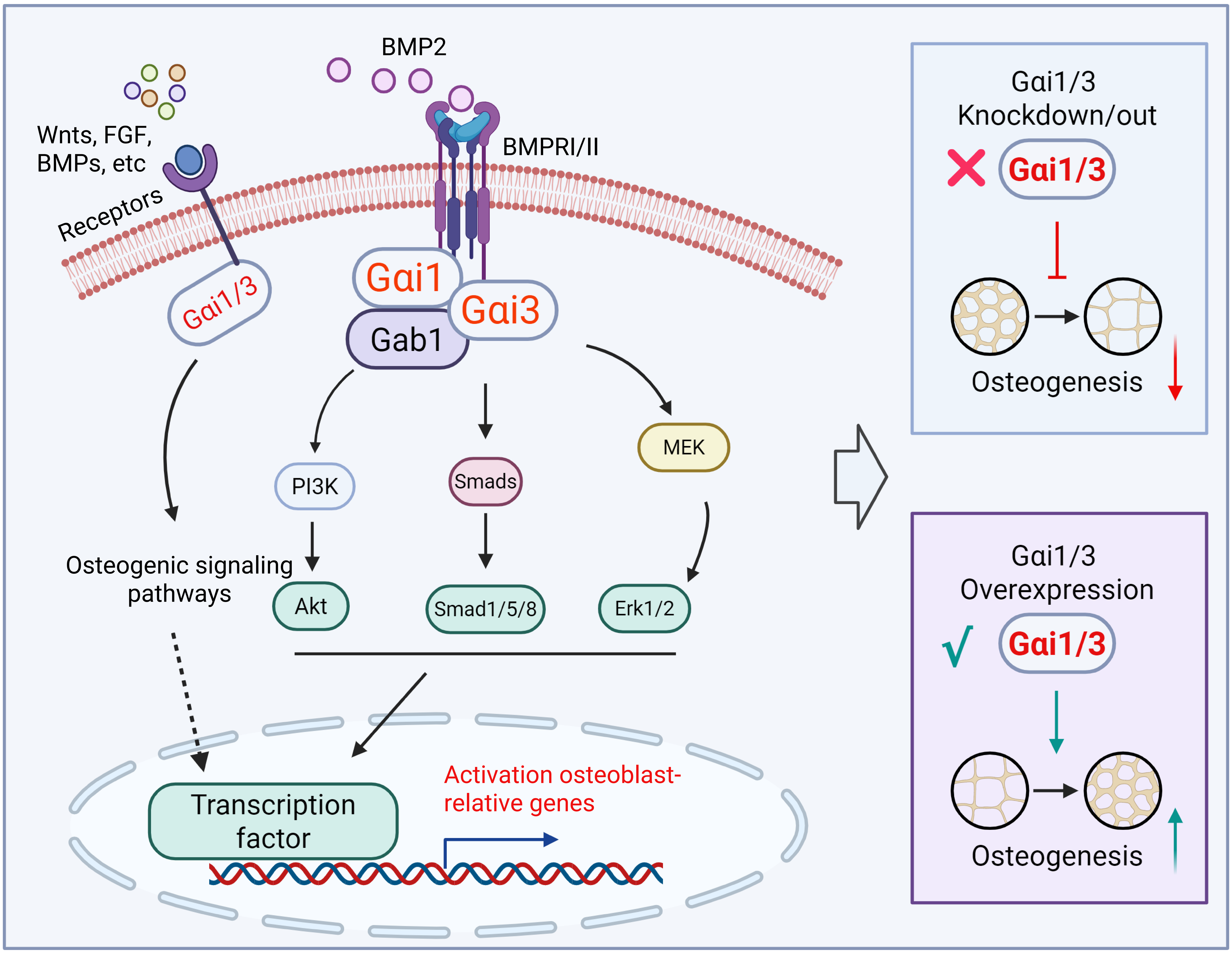

BMP2
Wnts, FGF, BMPs, etc
BMPRI/II
Receptors
Gαi1/3
Gαi1
Gαi3
Gab1
PI3K
Smads
MEK
Osteogenic signaling pathways
Akt
Smad1/5/8
Erk1/2
Transcription factor
Activation osteoblast-relative genes
Gαi1/3 Knockdown/out
Gαi1/3
Osteogenesis
Gαi1/3 Overexpression
√
Gαi1/3
Osteogenesis
