# Supplementary material for: Gαi1/3 Are Essential for BMP2‐Induced Osteogenesis by Bridging BMP Receptors to the Gab1 Signaling Complex
Source: Adv Sci (Weinh). 2026 Aug 10;13(59):e76837. doi: 10.1002/advs.76837 (PMC13454971; doi:10.1002/advs.76837)
Supplement: Supplementary file 2 [file ADVS-13-e76837-s001.docx]

**Figure S1. Overexpression of Gαi1/3 enhances the osteogenic effect of old BMSCs.** Extracted BMSCs from young and old mice. Old BMSCs were transfected the lentivirus Gαi1/3 construct (OE-Gαi1/3). Mineralization (ALP and Alizarin Red staining) and quantification measured at d21 (A-B). Statistical significance was obtained by Student’s t-tests. ∗∗*p <*0.01; ∗∗∗*p <*0.001. Data are means ± SD.

**Figure S2. Overexpression of Gαi1/3 restores the osteogenic capacity of Gαi1/3-DKO BMSCs.** BMSCs isolated of from WT and Gαi1/3 DKO mice. Gαi1/3-DKO BMSCs were transfected the lentivirus Gαi1/3 construct (OE-Gαi1/3). Mineralization (ALP and Alizarin Red staining) and quantification measured at d21 (A-B). Statistical significance was obtained by Student’s t-tests. ∗∗*p <*0.01; ∗∗∗*p <*0.001. Data are means ± SD.

**Figure S3. The construction of** **Gαi1/3-CKD and Col1a1-Cre; Gαi1/3^fl/fl^ mice.** Schematic of the AAV9 vector components (A). Western blot analysis and quantitative analysis of Gαi1 and Gαi3 in BMSCs (B). Breeding strategy of Col1a1-Cre; Gαi3^fl/fl^ mice (C). qPCR analysis of Gαi1 and Gαi3 in Col1a1-Cre; Gαi3^fl/fl^ osteoblasts (D). The expression patterns of Gαi2 mRNA in different types of mice. n=5 per group (E). The ratio of RANKL/OPG in aged male WT and DKO mice (F). n=5 per group. Scale bar: 50μm. All bar graphs are presented as the mean ± SD. ns, p > 0.05; ****p <* 0.001.

**Figure S4.** **Overexpression of Gab1 cannot restore the signal transduction disorder and osteogenic function limitation caused by Gαi1/3 deficiency.** Gαi1/3 DKO BMSCs were transiently transfected with the adenovirus Gab1 construct (“Ad-Gab1”), the adenovirus Gαi3 construct (“Ad-Gab1”) or the empty vector (“Vector”), Different groups were cultured DMEM medium supplemented with BMP2 (20 ng/ml) for indicated time, the proteins listed were examined by western blotting and phosphorylation (A). Osteoblast differentiation was induced by medium containing or without BMP2. Mineralization (ALP and Alizarin Red staining) and quantification measured at d21 (B-C). n = 3 per group. All bar graphs are presented as the mean ± SD. ns, p > 0.05.

**Figure S5. Gαi1 ~~had~~ binding sites with BMR1A/BMR1B/Gab1.** The Gαi1 protein is represented as a slate cartoon model, BMR1A / BMR1B/Gab1 protein is shown as a cyan cartoon model, and their binding sites are shown as the corresponding-colored stick structure. When focusing on the binding region, the binding site is then shown as a presentation of the protein to which it belongs (A-C). BMSCs were treated with BMP2 (20 ng/mL) for 10, 30 min, BMPRs-Gαi1-Gab1 association (“IP”) and expression (“Input”) was shown (D).

**Figure S6. The pattern of Gαi1/3-Gab1 plays a crucial role in bone formation.** Gαi1/3 protein is a key linker protein for the signal transduction of osteogenesis-related cytokines. Gαi1/3 mediates downstream signaling pathways such as Smads and MAPK by binding to adaptor proteins like Gab1, thereby promoting the osteogenic effect of BMSCs. Deficiency of either Gαi1/3 or Gab1 will inhibit the signal transduction of osteogenic factors and bone formation.
